# Systematic Review of Parameters of Stimulation, Clinical Trial Design Characteristics, and Motor Outcomes in Non-Invasive Brain Stimulation in Stroke

**DOI:** 10.3389/fpsyt.2012.00088

**Published:** 2012-11-12

**Authors:** Bamidele O. Adeyemo, Marcel Simis, Debora Duarte Macea, Felipe Fregni

**Affiliations:** ^1^Laboratory of Neuromodulation, Spaulding Rehabilitation Hospital, Harvard Medical SchoolBoston, MA, USA; ^2^Department of Physical Medicine and Rehabilitation, Spaulding Rehabilitation Hospital, Harvard Medical SchoolBoston, MA, USA; ^3^Division of Neurology, Santa Casa de São Paulo Medical SchoolSão Paulo, Brazil; ^4^Hospital das Clínicas da Faculdade de Medicina, University of São PauloSão Paulo, Brazil; ^5^Department of Neurology, Beth Israel Deaconess Medical Center, Harvard Medical SchoolBoston, MA, USA

**Keywords:** transcranial direct current stimulation, repetitive transcranial magnetic stimulation, stroke, motor, transcranial magnetic stimulation, noninvasive brain stimulation

## Abstract

**Introduction/Objectives:** Repetitive transcranial magnetic stimulation (rTMS) and transcranial direct current stimulation are two powerful non-invasive neuromodulatory therapies that have the potential to alter and evaluate the integrity of the corticospinal tract. Moreover, recent evidence has shown that brain stimulation might be beneficial in stroke recovery. Therefore, investigating and investing in innovative therapies that may improve neurorehabilitative stroke recovery are next steps in research and development. **Participants/Materials and Methods:** This article presents an up-to-date systematic review of the treatment effects of rTMS and tDCS on motor function. A literary search was conducted, utilizing search terms “stroke” and “transcranial stimulation.” Items were excluded if they failed to: (1) include stroke patients, (2) study motor outcomes, or (3) include rTMS/tDCS as treatments. Other exclusions included: (1) reviews, editorials, and letters, (2) animal or pediatric populations, (3) case reports or sample sizes ≤2 patients, and (4) primary outcomes of dysphagia, dysarthria, neglect, or swallowing. **Results:** Investigation of PubMed English Database prior to 01/01/2012 produced 695 applicable results. Studies were excluded based on the aforementioned criteria, resulting in 50 remaining studies. They included 1314 participants (1282 stroke patients and 32 healthy subjects) evaluated by motor function pre- and post-tDCS or rTMS. Heterogeneity among studies’ motor assessments was high and could not be accounted for by individual comparison. Pooled effect sizes for the impact of post-treatment improvement revealed consistently demonstrable improvements after tDCS and rTMS therapeutic stimulation. Most studies provided limited follow-up for long-term effects. **Conclusion:** It is apparent from the available studies that non-invasive stimulation may enhance motor recovery and may lead to clinically meaningful functional improvements in the stroke population. Only mild to no adverse events have been reported. Though results have been positive results, the large heterogeneity across articles precludes firm conclusions.

## Introduction

Stroke is a leading cause of disability in the United States. According to the American Heart Association, over 795,000 people experience strokes annually in the USA, with 185,000 presenting as recurrent strokes. Restitution of post-stroke motor function is frequently incomplete, with the majority of stroke patients unable to perform professional duties or activities of daily living by 6 months after their stroke. This becomes a self-fulfilling cycle of disability, as the decreased functional capacity predisposes toward deconditioning (or decreased physical activity) resulting in worsening cardiovascular disease and subsequent strokes (Hankey et al., 2002; Ivey et al., 2006).

The better understanding of plastic (or brain remodeling) changes following stroke have contributed to the development of novel targeted therapies that can modulate neuroplasticity, especially non-invasive methods such as transcranial magnetic stimulation (TMS) and transcranial direct current stimulation (tDCS).

One important finding is the notion that plasticity is not always adaptive. Therefore, therapies that block any potential maladaptive plasticity may be desirable. Specifically, several studies show the influence of maladaptive plasticity in sustaining behavioral deficits in stroke. For instance, neuroimaging analyses of stroke subjects have noted critical increases in cortical excitability in the intact primary motor cortex (M1) of the unaffected hemisphere (Hummel and Cohen, 2006), and this increased cortical excitability has been noted to correspond with movements of the paretic arm in patients with motor impairment (Calautti and Baron, 2003; Ward et al., 2003). In addition, the level of cortical excitability of the intact hemisphere directly correlates with the level of paresis in the affected extremity (Hummel and Cohen, 2006). Furthermore, post-stroke subjects exhibited changes in motor cortical excitability and abnormal levels of inter-hemispheric inhibition from the unaffected to the affected motor cortex (Hummel and Cohen, 2006). These observations have helped to develop the idea that there is maladaptive inter-hemispheric competition after stroke, which worsens hand paresis. Therefore, blocking or reducing maladaptive plasticity with neuromodulation techniques may be a desirable therapy as preliminary studies have shown. On the other hand, facilitatory stimulation may be provided to the affected hemisphere to enhance beneficial plasticity and improve motor outcomes (Hummel and Cohen, 2006).

Non-invasive procedures such as TMS and tDCS are elegant and powerful neuromodulatory techniques that create electric currents in the brain to change cortical excitability (Hummel and Cohen, 2006). TMS is a technique that induces a short electric pulse on the brain tissue via a varying magnetic field induced by the TMS coil, while tDCS reversibly polarizes brain regions through topical application of weak direct currents (Hummel and Cohen, 2006). Repetitive transcranial magnetic stimulation (rTMS) is a technique that provides continuous electric pulses on the brain in order to produce long-term changes in cortical excitability. Due to the relative focal target ability, safety profile, relative low cost, and positive preliminary results, these techniques have been extensively tested for the treatment of stroke.

In fact, recent studies have demonstrated that cortical brain stimulation achieved through invasive and non-invasive techniques improves motor function in stroke subjects. Small phase II trials have demonstrated that motor cortex stimulation with non-invasive techniques, rTMS and tDCS, can enhance motor function in stroke subjects significantly. The goal of this systematic review is to discuss the parameters of stimulation, clinical trial design characteristics, and evidence of effects from the available literature in the field. We (this research team) therefore reviewed clinical studies of rTMS and tDCS for motor recovery in stroke published in English from January 1st of 2002 to January 1st of 2012. We chose the period of 10 years in order to consider the most recent studies. We present our findings in the light of the state of the science and provide considerations and recommendations, with the aim of providing guidance for future studies.

## Methods

### Literature review

The first step of our systematic review was to perform a literature search utilizing the PubMed research database. Search strategy was implemented on PubMed to achieve higher standardization of results (Wong et al., 2006). In addition, we examined reference lists of the retrieved articles and consulted experts in the field. We performed a literary search utilizing the search terms “stroke” and “transcranial stimulation,” prior to (but not including) 01/01/2012, which resulted in 695 articles. Individual search terms were used instead of mesh terms in order to increase the number of results retrieved. We added the search term “motor” to our search, which produced 513 articles. We further elucidated the results by performing two sub-search inclusions: (1) the first added the key search terms “repetitive”; (2) while the other added the search term “direct,” resulting in 142 articles and 74 articles, respectively. We also cross-reference checked by using the terms “neurostimulation” and the acronyms “rTMS” and “tDCS” in lieu of their spelled-out counterparts. We found a total of 201 articles related to the use of repetitive transcranial current stimulation or tDCS in stroke patients to evaluate motor outcomes. We subsequently checked each article according to our inclusion criteria.

### Selection criteria

We included prospective studies that evaluated the effects of a treatment with rTMS and tDCS on the motor rehabilitation of patients with non-hyperacute strokes. We adopted the following inclusion criteria: (1) articles written in English; (2) non-invasive brain stimulation techniques (rTMS and tDCS) for the recovery of motor impairments in patients with non-hyperacute stroke; (3) use of scales to measure motor recovery; (4) studies published in a book, journal, proceeding, or indexed abstraction; (5) studies reporting the motor recovery scale before and after the treatment; (6) studies published with the 10-year period; and (7) treatments that included neuromodulation techniques as the main strategy to treat motor impairments in stroke. Items were excluded if they failed to (1) include stroke patients, (2) study motor outcomes, or (3) include rTMS/tDCS as treatments. Other exclusions included (1) reviews, editorials, or letters (2) animal or pediatric populations, (3) case reports or sample sizes ≤2 patients, (4) primary outcomes of dysphagia, dysarthria, neglect, or swallowing.

### Data extraction

The data were extracted by two authors (Bamidele O. Adeyemo and Debora Duarte Macea), using a structured form, and checked by another author (Marcel Simis). The following variables were extracted: (1) mean and SD of motor scales before and after treatment and at follow-up (when available) for the active and control groups; (2) demographic, clinical, and treatment characteristics (e.g., number of patients in the control and treatment groups, age, gender, baseline characteristics, region of stroke, type of stroke, post-injury duration, stroke severity, history of previous stroke, baseline motor function, and strength/spasticity); (3) intervention protocol type; (4) rTMS stimulation parameters (TMS type, target muscles, type of coil, frequency, intensity-%motor threshold, number of stimuli per train, inter-train interval, and number of trains); (5) tDCS stimulation parameters [intensity, duration, location, electrode (info and size)]; (6) concomitant treatments (therapy and medications); (7) methods of assessment; and (8) evaluation model and design. When a study did not report the SD for motor outcomes, we deduced them from other parameters, contacted the authors, or made note as to their availability.

### Quantitative analysis and statistical analysis

All of our analyses were performed utilizing STATA statistical software, version 8.0 (StataCorp, College Station, TX, USA). We initially computed the standardized mean difference and the pooled SD for each comparison. Given the heterogeneous motor outcomes, we focused the additional analysis to the statistically significant reports available in the article. We utilized Cohen’s *d* as an appraisal of the effect size, which was calculated by comparing pre and post-treatment mean changes of the treatment groups. Subsequently, we computed the pooled weighted effect size (weighted by the inverse variance of each study), utilizing random and fixed effect models. The random effect model lends relatively more weight to smaller studies and wider confidence intervals than the fixed effect model.

We also assessed publication bias utilizing the Begg-modified funnel plot. This figure plotted the standardized mean difference of each plot on a logarithmic scale against the respective standard error per study. We also applied the Egger’s test to evaluate for any significant asymmetry. The Egger test helps identify publication bias as follows: in scenarios where the effects from the smaller studies differ from the effects reported in the larger studies, the regression line will fail to run through the origin. This might indicate publication bias where smaller studies with negative results are not published (Egger et al., 1997).

## Results

Our study includes 10-year data prior to 01/01/2012 of randomized clinical trials, assessing 1314 subjects (1282 stroke patients and 32 healthy subjects). The results of this systematic review suggest that the use of non-invasive brain stimulation interventions in patients with stroke are associated with improvements in motor outcomes both individually and when compared to placebo stimulation. The 50 studies showed a large variability in the type of assessments that were used, the study population, the etiology and characteristics of the stroke, and time of intervention.

### Studies retrieval

Keyword searches on the PubMed database yielded 695 citations. Using our study criteria, we narrowed the list to 201 citations. Using our inclusion criteria, 50 articles met all our criteria and were analyzed in our review. Keyword searches on the PubMed database yielded 695 citations. Using our study criteria, we narrowed the list to 201 citations. Using our inclusion criteria, 50 articles met all our inclusion criteria and were analyzed in our review. References were excluded for (1) being non-English (narrowing to 201 citations) (2) editorial/s, review/s, letters, animal, pediatric, case reports, dysphagia, dysarthria, neglect, or swallowing (narrowing to 131 citations) (2) including the term repetitive but not related to rTMS (117 citations remaining) (3) use pain rather than motor outcomes (107 remaining citations) (4) employ theta burst or Hebbian montage (101 remaining citations) (4) not studying stroke subjects or having publication dates prior to 01/01/2012, totaling 50 meeting inclusion criteria.

### Demographic findings

Aggregation of participant data demonstrated a total of 1282 stroke patient participants (37% women) and the average per study was 26.04 participants. The average age of the participants was 58.46 (range of 18–95) years. (Note: the article, Lomarev et al. (2007) was not included in the average because it did not provide the necessary data to calculate average.) Demographic findings of these studies are summarized in Table 1.

**Table 1:** **Baseline demographic characteristics of all selected peer-reviewed articles**.

Reference	Number of subjects	Age-mean	Age-SD	Cortical/subcortical	Hemorrhagic versus ischemic	Stroke severity (mild/mod/severe)	Females (%)	Stroke duration (months)	Oxford quality scoring system
Werhahn et al. (2003)	General: 20 stroke; 10 healthy	61.5	13.6	5 Cortical; 14 subcortical (2 in pons); 1 corticosubcortical	Ischemic	Mixed based MRC (1–4)	30% Healthy 40%	74.40	1
Takeuchi et al. (2005)	20	59	9.6	20 Subcortical	Ischemic	Mixed. Based FM (25–100)	25%	26.95	2
Mansur et al. (2005)	10 stroke; 6 healthy	53.3	X	3 Cortical and 7 subcortical	Ischemic	Mixed, hemiparesis 1 subtle/4 mild/3 moderate/1 severe/1 not specified (2 least excluded)	70%	X	1
Khedr et al. (2005)	52	52.85	rTMS group: 9.5; sham group: 8.4	Cortical 15; subcortical 26; corticosubcortical 11	Ischemic	Moderate to severe based NIHSS	31%	7.20	0
Fregni et al. (2005)	6	53.7	16.60	Cortical 1; subcortical 3; corticosubcortical 2	NA	Mild to moderate based on MRC (3.5–4.5)	67%	27.10	1
Fregni et al. (2006)	15	57.7	11.27	2 Cortical/13 subcortical	Ischemic	Mild to moderate motor deficit	27%	44.05	2
Lotze et al. (2006)	7 Stroke; 7 healthy	63.7	8.6	Subcortical	Ischemic	Severely paretic or even hemiplegic at their first day after stroke, four in their left hand and three in their right hand. Almost complete recovery of motor function	14%	33.90	0
Hummel and Cohen (2006)	11	57	16.00	Mostly subcortical	ischemic	Severe upper arm motor paresis (below MRC grade 2). Some of them remained unable to complete the Jebsen–Taylor Test	55%	41.80	2
Richards et al. (2006)	19	60.12	15.1	Cortical, subcortical, and brain stem	Ischemic and hemorrhagic	Mixed WMTF 19.17 (SD: 18.8)	8	81.60	3
Kim et al. (2006)	15	53.5	4.5	Cortical 5 and subcortical 10	3 Hemorrhagic, 12 ischemic	Mild to moderate rankin (1–3)	13%	16.70	1
Liepert et al. (2007)	12	63	11	Subcortical 12(2 pons)	NA	Mild based MRC (4)	33%	0.24	1
Lomarev et al. (2007)	7	X	X	2 Subcortical 5 corticosubcortical	I:6 × H:1	Sufficient residual motor function in the paretic arm to perform pinch test dynamometry (range 25.1–31.0 lb). Patients unable to extend at the metacarpophalangeal joints at least 10–20° were also excluded from the study	29%	X	0
Malcolm et al. (2007)	19	67	6.8	NA/NA/mixed(11 MCA; 7 lacunar; 1 unlisted); cortico + subcortical	1 Hemorrhagic; 18 ischemic	Mixed based WMFT 15.5 ± 13.1 rTMS; 35.5 ± 33.9 sham	42%, 8	45.60	2
Hesse et al. (2007)	10	63.3	X	8 Cortical; 2 subcortical	Ischemic	Severe arm paresis	70%	X	3
Boggio et al. (2007)	9	57.4	12.9	Subcortical	NA	Mild-moderate based on MRC (3.7–4.8)	22%	40.90	1
Pomeroy et al. (2007)	27	74.8	12.71	Cortical 8; subcortical 17; corticosubcortical 2	Ischemic	Had upper limb weakness due to the stroke but able to produce at least a voluntary twitch of paretic biceps and/or triceps	67%	0.89	3
Nowak et al. (2008)	15	460,667	8.03	15 Subcortical	Ischemic	Mild based MRC (4–5)	27%	1.93	1
Takeuchi et al. (2008)	20	62.3	8.04	Subcortical	Ischemic	Mixed. based FM (33–91)	20%	29.90	2
Dafotakis et al. (2008)	12	45	9.00	Subcortical	Ischemic	Mild based MRC (4–5)	33%	1.88	0
Mally and Dinya (2008)	64	57.6	10.8	Cortical – large hemispheric lesion	46 Ischemic, 18 hemorrhagic	Severe	42%	129.60	0
Yozbatiran et al. (2009)	12	67	12.00	Cortical 1; subcortical 11	Ischemic, or hemorrhagic but not subarachnoid	(1) Arm motor FM score 15–55 out of 66. (2) moderate–severe arm motor deficits	17%	4.10	0
Ameli et al. (2009)	29	56	13.00	Cortical 13; subcortical 16	Ischemic	Mild-moderate	45%	5.50	0
Khedr et al. (2009)	36	57.9	11.00	Cortical 19 and subcortical 17	Ischemic	Mild to moderate	47%	0.57	1
Takeuchi et al. (2009)	30	59.3	12.4	Subcortical	Ischemic	Mixed based FM	27%	28.80	2
Kakuda et al. (2010b)	15	55	17	Subcortical 3 (1 pons); corticosubcortical 2	Hemorrhagic 9 × ischemic 6	Moderate to severe based FM (17–57)	33%	57.00	0
Grefkes et al. (2010)	11	46	9.3	Subcortical	Ischemic	Mild based MRC (4–5)	18%	1.91	1
Khedr et al. (2010)	48	59.52	13.1	Cortical 13; Subcortical 35	Ischemic	Mixed based NIHSS	50%	0.22	2
Emara et al. (2010)	60	53.9	X	Cortical 22; Subcortical 38	Ischemic	Mild to moderate hand weakness	33%	4.16	1
Lindenberg et al. (2010)	20	58.75	14.07/12.9 (sham)	Corticosubcortical (medial cerebral artery)	Ischemic	Severe based FM (20–56)	20%/30% (sham)	35.40	3
Chang et al. (2010)	28	56.6	12.2	Cortical 11; subcortical 17 (6 pons, 2 medial medullar)	Ischemic	Mixed; mild to severe	39%; 11	0.45	2
Kim et al. (2010b)	18	57.8	X	Cortical 5; subcortical 9; corticosubcortical 4	Ischemic	Mixed based MRC (2–5) and FM (16–60)	5	0.85	3
Kakuda et al. (2010a)	5	66.8	X	subcortical	1 Hemorrhagic, 4 ischemic	Mild to moderate rankin (1–3)	2	36.60	0
Koganemaru et al. (2010)	9 Stroke + 9 Healthy	51.6 rTMS; 53.2 healthy	11.6 rTMS; 13.8 healthy	Subcortical (1 pons)	Ischemic 7; hemorrhage 2	Mixed based: Stroke Impairment Assessment Set (SIAS)	55%, 5 (both)	24.00	1
Kakuda et al. (2011b)	39	56.5	16.0	Not specified	H:23 (59); I: 16 (41)	NA (FM 36 average)	23%	50.30	0
Kakuda et al. (2011d)	52	57	13	Cortical and subcortical	Hemorrhagic 30 × ischemic 22	Brunnstrom Stage 3–5	14 (27%)	52.40	0
Kakuda et al. (2012)	204	58.5	13.4	Cortical and subcortical	hemorrhagic 107 × ischemic 97	Brunnstrom Stage 3–5	73 (36%)	60.00	0
Sasaki et al. (2011)	29	66.96		supratentorial subcortical	Hemorrhagic 16 × ischemic 13	NIHSS = 6.29	9 (31%)	0.56	1
Stagg et al. (2012)	1) 13; 2) 11(Note: 7 in both; 17 total)	64 years	Range 30–80 years	Cortical and subcortical	Hemorrhage 1; ischemic 16	Not mentioned	4 (23.5%)	37.90	1
Kakuda et al. (2011c)	11	61	13.7	Subcortical	hemorrhage 7; Ischemic 4	Brunnstrom Stage 3–5	5(45.4%)	69.90	0
Madhavan et al. (2011)	9	65.4	13.2 (50–87 years)	Cortical and subcortical	Not mentioned	Lower extremity Fugl-Meyer 21–30 (maximum score 32)	4(44.4%)	130.80	0
Tanaka et al. (2011)	8	59.6	3.9	Subcortical	Not mentioned	Mixed based SIAS	4(50%)	21.10	1
Kakuda et al. (2011a)	5	61	56–66	Subcortical	Hemorrhage 4; ischemic 1	Brunnstrom Stage 3–5	2(40%)	64.00	1
Avenanti et al. (2012)	30	60.9 rTMS-PT; 64.0 PT-rTMS; 64.0 sham	8.8 rTMS-PT; 7.7 PT-rTMS; 12.1 sham	Cortical 3; corticosubcortical 1; subcortical 26	Hemorrhage 10; ischemic 20	Mild severity based on inclusion criteria	47%	31.47	3
Bolognini et al. (2011)	14	46.71	14.08	Cortical 9; corticosubcortical 5	Ischemic 12; hemorrhagic 2	Moderate to severe hemiparesis, per Fugl-Meyer (Stroke duration 35.21 ± 26.45)	64%	35.21	2
Conforto et al. (2012)	30	54.8 rTMS, 56.7 sham	11.7 rTMS, 14.8 sham	16 Subcortical, 14 cortical	Ischemic	Mixed; mild to sever per NIHSS (range 1–11) and FM (50–123)	40%	0.92	3
Hesse et al. (2011)	96	63.9 anodal tDCS; 65.4 cathodal tDCS; 65.6 sham	10.5 anodal tDCS; 8.6 cathodal tDCS; 65.6 sham	Mixed (cortical or corticosubcortical): 25 anodal tDCS, 24 cathodal tDCS, 26 sham tDCS. Subcortical 7 anodal tDCS, 8 cathodal tDCS, 6 sham	Ischemic	Severe per Upper extremity FM (7.8 ± 3.8 anodal tDCS, 7.9 ± 3.4 cathodal tDCS, 8.2 ± 4.4 sham; Stroke duration in weeks 3.4 ± 1.8 anodal tDCS, 3.8 ± 1.4 cathodal tDCS, 3.8 ± 1.5 sham)	39%	0.93	3
Mahmoudi et al. (2011)	10	60.8	14.1	Cortical 7, subcortical 3	Ischemic	Mild to moderate deficit (based on patients’ ability to perform all items of			
						Jebsen–Taylor Test (JTT)	30%	8.30	1
Nair et al. (2011)	14	55.8	(Range 40–76)	Cortical 9, subcortical 5	NA	Moderate to severe upper extremity impairment [per upper extremity Fugl-Meyer of 30.1 (±10.4)]	36%	30.50	2
Chang et al. (2012)	21	58.1 rTMS; sham 59.5	9.75 rTMS; 11.40 sham	Cortical 2, subcortical 15	3 Hemorrhagic, 14 ischemic	NA	41%	10.06	3
Hummel et al. (2005)	6	62.2	7.56	Subcortical	Ischemic	Mild (MRC4.8 ± 0.03)	33%	44.30	1

*The phrases ‘X’ and NA denote unavailable information*.

The number of studies seemed to be stable over this 10 year period (with an average of 4.9 studies per year), though it appears that there was an increase in the last 2 years (2010 and 2011) with a peak of 13 studies. The methodological quality of the articles was assessed utilizing the Oxford quality scoring system (Jadad scale). Scores range from 0 to 3 and are listed in Table 1 (Jadad et al., 1996; Olivo et al., 2008).

The average of the stroke duration (time after stroke) of the patients in the selected articles was 33.03 months. The individual values are represented in Table 1. Most of the articles included patients in the chronic stroke phase. There are six articles (Hesse et al., 2007, 2011; Dafotakis et al., 2008; Kim et al., 2010b; Sasaki et al., 2011; Conforto et al., 2012) that included subacute stroke phase and four articles (Liepert et al., 2007; Khedr et al., 2009, 2010; Chang et al., 2010) that were conducted in acute phase of the stroke. Other demographic characteristics are included in Table 1.

### Stroke characteristics

We identified two articles that did not specify when the stimulation was applied regarding the time course of the stroke (Pomeroy et al., 2007; Nowak et al., 2008). Most of the studies administered stimulation during the chronic phase, rather than acute or subacute. One issue here is the definition of chronic stroke that is not well defined, which is discussed further below. The selected studies included ischemic stroke only (49.0%), both ischemic and hemorrhagic stroke, or did not specify the type of stroke (as summarized in Table 1).

The predominant location of the stroke was cortical and subcortical [28 (56.0%)]; followed by subcortical only [15 (30%)], cortical, subcortical, and brain stem [4 (8.2%)], subcortical and brain stem [2 (4.1%)], and one article (2.9%) did not specify the location. There were no articles reporting patients with bilateral lesions.

Most of the studies included a heterogeneous population either including the full spectrum of severity (mild to severe – 11 studies (22.4%) or at least two of the three categories (mild to moderate or moderate to severe). In four articles, it was not possible to classify the severity (Richards et al., 2006; Lomarev et al., 2007; Pomeroy et al., 2007; Kakuda et al., 2011b; Chang et al., 2012; Stagg et al., 2012).

### Adjuvant therapies

Different types of therapies associated with the neuromodulation techniques as main intervention were used. The main therapies were Constraint Induced Movement Therapy (CIMT), robotic, and standard therapy (unspecified). They are listed in Tables 3 and 4.

### Motor outcomes

Different study designs and assessments employed in the evaluation of post-stroke motor function were used. The outcomes addressed the following: (1) motor function only; (2) safety and motor function; (3) motor function and fMRI data; (4) motor function and therapy; (5) motor function, fMRI, and therapy; and (6) motor function and voluntary muscle contraction. Specifically, we categorized all of the articles in Table 2 according to the motor assessment tool used. We also indicated which results were reported to be statistically significant. The articles assessed for motor strength, dexterity, range of motion, and disability. This information is delineated in Table 2.

**Table 2:** **Inventory of motor assessment tools and reported outcome in selected transcranial Stimulation articles**.

Reference	MRC	JTHF	HG	PF	PA	FM	PTT	sRT	cRT	MRs	NIHSS	FT	RGM	ASS	WMFT	ARAT	MA.Log	BBT	BIS	ASMI	LSMI	FAC	B	Others
Fregni et al. (2006)	B	# +	N	N	N	N	# +	# +	# +	N	N	N	N	N	N	N	N	N	N	N	N	N	N	N
Takeuchi et al. (2005)	N	N	N	Φ	+	N	N	N	N	N	N	N	N	N	N	N	N	N	N	N	N	N	N	N
Mansur et al. (2005)	N	N	N	N	N	N	+	+	+	N	N	Φ	N	N	N	N	N	N	N	N	N	N	N	N
Werhahn et al. (2003)	B	N	N	N	N	B	N	Δ	N	N	N	♦	N	N	N	N	N	N	N	N	N	N	N	N
Nowak et al. (2008)	B	N	N	N	N	N	N	N	N	B	B	+	+	N	B	N	N	N	N	N	N	N	N	N
Takeuchi et al. (2008)	N	N	N	# +	# +	B	N	N	N	N	N	N	N	N	N	N	N	N	N	N	N	N	N	N
Dafotakis et al. (2008)	B	N	N	+	+	N	N	N	N	B	B	N	N	N	N	B	N	N	N	N	N	N	N	N
Kakuda et al. (2010b)	N	N	N	N	N		N	N	N	N	N	N	N	*	*	N	N	N	N	N	N	N	B	N
Liepert et al. (2007)	B	N	Φ	N	N	N	Φ	N	N	N	N	N	N	N	N	N	N	N	N	N	N	N	N	N
Grefkes et al. (2010)	B	N	N	N	N	N	N	N	N	B	B	N	N	N	N	B	N	N	N	N	N	N	N	Written instructions on a monitor visible through a mirror whether to move the left, right, or both hands in the upcoming task-block (+)
Lotze et al. (2006)	B	N	N	N	N	N	N	N	N	N	N	Δ	N	N	N	N	N	N	N	N	N	N	N	N
Lomarev et al. (2007)	N	N	N	Φ	N	N	N	N	N	N	N	N	N	N	N	N	N	N	N	N	N	N	N	N
Malcolm et al. (2007)	N	N	N	N	N	N	N	N	N	N	N	N	N	N	Φ	N	Φ	Φ	N	N	N	N	N	N
Khedr et al. (2005)	N	N	N	N	N	N	N	N	N	N	# +	N	N	N	N	N	N	N	# +	N	N	N	N	SSS# +
Khedr et al. (2010)	N	N	*#	N	N	N	N	N	N	#	+	N	N	N	N	N	N	N	N	N	N	N	N	Shoulder abduction #; Dorsiflexion of toes Hip flexion#; Toe dorsiflexion# (only after 1 year fir 10 Hz)
Yozbatiran et al. (2009)	N	N	*	N	N	Ξ	*	N	N	N	N	N	N	N	N	Φ	N	N	Φ	N	N	N	N	Active ranges of motion at the affected side wrist and index finger metacarpophalangeal joint.
Ameli et al. (2009)	B	N	N	N	N	N	N	N	N	B	B	(+) And hand tapping +	N	N	N	B	N	N	N	N	N	N	N	N
Hummel and Cohen (2006)	B	N	N	+	N	B	N	+	N	N	N	N	N	B	N	N	N	N	N	N	N	N	N	N
Hesse et al. (2007)	*	N	N	N	N	*	N	N	N	N	N	N	N	N	N	N	N	N	N	N	N	N	N	N
Fregni et al. (2005)	B	+	N	N	N	N	N	N	N	N	N	N	N	B	N	N	N	N	N	N	N	N	N	N
Emara et al. (2010)	N	N	N	N	N	N	N	N	N	# +	N	+	N	N	N	N	N	N	N	N	N	N	N	Activity Index (AI) scale# +
Boggio et al. (2007)	B	# +	N	N	N	N	N	N	N	N	N	N	N	N	N		N	N	N	N	N	N	N	N
Pomeroy et al. (2007)	N	N	N	N	N	N	N	N	N	N	N	N	N	N	N	Φ	N	N	N	N	N	N	N	Peak torque about the elbow during isotonic concentric flexion/extension.
Lindenberg et al. (2010)	B	N	N	N	N	# + UE	N	N	N	N	N	N	N	N	# +	N	N	N	N	N	N	N	N	For fMRI, performing repetitive elbow and wrist extension/flexion movements
Chang et al. (2010)	N	N	# +	N	N	(1) UE + (2) LL	N	N	N	N	N	N	N	N	N	N	N	Φ	Φ	# +	Φ	Φ	Φ	N
Kim et al. (2010b)	B	N	N	N	N	#	N	N	N	B	B	N	N	N	N	N	N	N	Φ	N	N	N	B	N
Kakuda et al. (2011b)	N	N	N	N	N	Ξ *	N	N	N	N	N	N	N	Ξ*	*	N	N	N	N	N	N	N	N	N
Kakuda et al. (2010a)	N	N	N	N	N	Ξ *	N	N	N	N	N	N	N	N	Ξ *	N	N	N	N	N	N	N	B	Ten seconds test X *
Koganemaru et al. (2010)	N	N	(A) + (after 30 min); (B) Ξ *	(B) Ξ *	N	N	N	N	N	N	N	N	N	(A) +; (B) Ξ *	N	N	N	N	N	N	N	N	N	(A) Active range of movement +(B) active range of movement X *; passive range of movement X *
Richards et al. (2006)	N	N	N	N	N	N	N	N	N	N	N	N	N	N	Φ	N	N	N	N	N	N	N	N	Motor Activity Log.3
Kim et al. (2006)	N	N	N	N	N	N	N	N	N	N	N	N	N	N	N	N	N	N	N	N	N	N	N	Finger motor task. (1) Movement accuracy (MA) + (2) movement time (MT) +
Khedr et al. (2009)	N	N	Φ	N	N	N	# +	N	N	N	# +	# +	N	N	N	N	N	N	# +	N	N	N	N	Keyboard tapping#*
Takeuchi et al. (2009)	N	N	N	# +	# +	N	N	N	N	N	N	N	N	N	N	N	N	N	N	N	N	N	N	N
Mally and Dinya (2008)	N	N	N	N	N	Ξ *	N	N	N	N	N	N	N	N	N	N	N	N	N	N	N	N	N	Score of spasticity at rest X *
Kakuda et al. (2011d)	N	N	N	N	N	*	N	N	N	N	N	N	N	N	*	N	N	N	N	N	N	N	B	N
Kakuda et al. (2012)	N	N	N	N	N	Ξ *	N	N	N	N	N	N	N	N	Ξ *	N	N	N	N	N	N	N	B	N
Sasaki et al. (2011)	N	N	+	N	N	N	N	N	N	N	B	+	N	N	N	N	N	N	N	N	N	N	B	N
Stagg et al. (2012)	N	N	(1) Φ	N	N	N	N	(2) (+)	N	N	N	N	N	N	N	N	N	N	N	N	N	N	N	(1) Response times (+); (2) choice response time condition (Φ).
Kakuda et al. (2011c)	N	N	N	N	N	*	N	N	N	N	N	N	N	N	*	N	N	N	N	N	N	N	B	N
Madhavan et al. (2011)	N	N	N	N	N	B	N	N	N	N	N	N	N	N	N	N	N	N	N	N	N	N	N	Tracking a sinusoidal waveform (+)
Tanaka et al. (2011)	N	N	Φ	N	N	N	N	N	N	N	N	N	N	N	N	N	N	N	N	N	N	N	N	SIAS (B); MFKE(+)
Kakuda et al. (2011a)	N	N	N	N	N	* Ξ	N	N	N	N	N	N	N	*	* Ξ	N	N	N	N	N	N	N	N	N
Avenanti et al. (2012)	N	+#	=#	PG# +; force +	N	N	N	N	N	N	N	N	N	N	N	N	N	+#	N	N	N	N	N	NHPT +#
Bolognini et al. (2011)	N	+#	+	N	N	+	N	N	N	N	N	N	N	N	N	N	+#	N	N	N	N	N	N	N
Conforto et al. (2012)	N	+#	N	+	N	+	N	N	N	+	B	N	N	Φ	N	N	N	N	N	N	N	N	N	N
Hesse et al. (2011)	+#	N	N	N	N	+#	N	N	N	N	N	N	N	+#	N	N	N	+#	+#	N	N	N	N	N
Mahmoudi et al. (2011)	N	+	N	N	N	N	N	N	N	N	N	N	N	N	N	N	N	N	N	N	N	N	B	
Nair et al. (2011)	N	N	N	N	N	+	N	N	N	N	N	N	N	N	N	N	N	N	N	N	N	N	N	Mean ROM shoulder abduction, elbow extension and wrist extension +
Chang et al. (2012)	N	+	N	N	N	N	N	N	N	N	N	N	N	N	N	N	N	N	N	N	N	N	N	Sequential motor task – accuracy (MA) +
Hummel et al. (2005)	B	+	N	N	N	B	N	N	N	N	N	N	N	B	N	N	N	N	N	N	N	N	N	Hand ability B

JTHF, Jebsen–Taylor hand function test; MRC, Medical Research Council scale; HG, hand grip; PF, pinch force; PA, pinch acceleration; FM, Fugl-Meyer assessment, PTT, Purdue Pegboard test; sRT, simple reaction time test, cRT, choice reaction test; MRs, modified rankin score; NIHSS, National Institutes of Health Stroke Scale; FT, finger tapping (with different tasks); RGM, reach to grasp movement, ASS, Ashworth spasticity scale; WMFT, wolf motor function test; ARAT, action research arm test; MA.Log, motor activity log; BBT, box and block test; BIS, Barthel index scale; ASMI, arm score of motricity index; LSMI, leg score of motricity index; FAC, functional ambulatory category; B, Brunnstrom; SSS, Scandinavian stroke scale; UE, upper extremity; LL, lower limb; SIAS, stroke impairment assessment set; MFKE, maximal force of knee extension. (B) = apply in baseline only; (Δ) = negative; (Φ) = no significant change; (*) = positive, but no placebo control; (+) = positive; (#) = long-term positive; (Ξ) = long-term positive, but placebo control; (♦) = affect.

### Adverse effects of non-invasive stimulation

There was a large heterogeneity in the reporting of safety including different safety assessment tools and inclusion/exclusion criteria. There were no significant major safety events in the selected studies. Neurocognitive assessments as an index for safety were conducted in only a few of the studies (Fregni et al., 2006; Emara et al., 2010). None of the selected articles investigated mood changes following stimulation. Some of the articles have considered psychiatric illness as exclusion criteria (see Table S1 in Supplementary Material).

No major adverse effects have been reported. The side effects reported were tingling, headache, dizziness, itching, and increase in anxiety. In Fregni et al. (2006), one patient in the sham rTMS group reported an increase in the tiredness and another one noted a mild headache (Fregni et al., 2006).

Yozbatiran et al. (2009) showed a change in blood pressure of 7 mm Hg when assessing the effects of rTMS. We have noticed a variability of adverse effects in the articles. For the articles that did not specifically mention side effect, it should be noted absence of report does not imply absence of effect. These results are summarized in Table S1 in Supplementary Material.

Other measures of safety were used such as electroencephalography (EEG), which was as an exclusion criteria or a safety outcome. Studies using EEG as outcomes showed no changes in EEG post stimulation (Table S1 in Supplementary Material). Although rare, some subjects had dropped out of the studies because of adverse events. In Lomarev et al. (2007), one subject dropped out for not being able to tolerate the rTMS train at 100%. In Kim et al. (2010b), two patients discontinued treatment with tDCS; one due to headaches and the other due to dizziness. In Stagg et al. (2012), two patients withdrew from the study before completion: one due to claustrophobia and the other due to unrelated medical reasons. Both were noted to be unrelated to tDCS. These results are further listed in Table S1 in Supplementary Material.

### Effects of gender on brain stimulation and stroke population

There was significant variability in number of male versus female patients in the selected articles. Information of individual analysis of motor effect per patient gender was unavailable for comparison. Therefore, aggregate analysis was conducted utilizing gender percentages (Table 1) per motor effect size. The mean male:female ratio was 63:37% of stroke patients in the selected articles. The analysis failed to find significant correlation; however there was a slightly positive trend for increased effect size as male percentage increased (*y* = 1.0257*x* − 0.0117. *R*^2^ = 0.0646) and a conversely decreased correlation of effect size where the percentage of females were higher (*y* = −1.0257*x* + 1.014, *R*^2^ = 0.0646).

### Stimulation parameters and protocol

On review of selected articles, 36 (72.0%) used TMS as intervention, while 14 (28.0%) of the articles used tDCS stimulation. Most of the articles were designed with a strategy to decrease the contralateral hemisphere or increase the activity in the ipsilesional hemisphere (usually by increasing the activity of the peri-lesional area). Some articles utilized both paradigms. One important exception for this approach is the study by Mally and Dinya (2008) that demonstrated motor improvement by inhibiting the peri-lesional region. However, it is also important to note that there was no placebo control included here. We have summarized the different protocols in Tables 3 and 4.

**Table 3:** **Parameters for TDCS application parameters, placement, electrode Attributes, and employed placebo method**.

Reference	Intensity (mA)	Duration (min)	Number sections	Location anode	Location cathode	Electrode (info and size)	Type of placebo	Concomitant therapy or motor tasks
Hummel and Cohen (2006)	1	20	1	Motor ipsilesional side	Contralateral Supraorbital	25 cm^2^	30 s	N/A
Hesse et al. (2007)	1.5	7	30	Motor ipsilesional side	Contralateral Supraorbital	35 cm^2^	No sham	Robot-assisted arm training
Fregni et al. (2005)	1	20	1	Ipsilesional side Or Supraorbital	Contralesional or supraorbital	35 cm^2^	30 s	N/A
Boggio et al. (2007)	1	20	(1) 1 and (2) 5	(1) Ipsilesional side or supraorbital; (2) Supraorbital	(1) Contralesional side or supraorbital (2) contralesional side	35 cm^2^	30 s	N/A
Lindenberg et al. (2010)	1.5	30	5	Motor ipsilesional side	Motor contralesional side	16.3 cm^2^	30 s	Physical and occupational therapy
Kim et al. (2010b)	2	20	10	Ipsilesional side Or Supraorbital	Contralesional or Supraorbital	25 cm^2^	60 s	Conventional, physical, and occupational therapy
Stagg et al. (2012)	1	(1) 20 (2) 10	1 session (3 total crossover)	Motor ipsilesional side or supraorbital	Motor contralesional side or supraorbital	35 cm^2^	10 s (vertex)	N/A
Madhavan et al. (2011)	0.5	15	1 session (3 total crossover)	Motor ipsilesional side or contralesional (Leg)	Contralateral Supraorbital	8 cm^2^ (anode) 48 cm^2^ (cathode)	10 s	N/A
Tanaka et al. (2011)	2	10	1 session (2 total crossover)	Motor ipsilesional side (Leg)	Contralateral Supraorbital	35 cm^2^ (anode) 50 cm^2^ (cathode)	15 s	N/A
Bolognini et al. (2011)	2	40	10	Ipsilesional side	Contralesional side	35 cm^2^	30 s	Constraint-induced movement therapy
Hesse et al. (2011)	2	20	30	Group A: ipsilesional side; Group B: contralesional side; sham: A or B set-up changing consecutively	Group A: contralateral orbit; Group B: contralateral supraorbit area; Sham: A or B set-up changing consecutively	35 cm^2^	0 mA	Robot-assisted arm training
Mahmoudi et al. (2011)	1	20	1 session (5 total crossover)	Bilateral tDCS group: ipsilesional side; Anodal tDCS group: ipsilesional side; Cathodal tDCS group: contralateral supraorbital area; Extra-cephalic tDCS group: ipsilesional side; Sham tDCS group	Bilateral tDCS group: contralesional side; Anodal tDCS group: Contralateral supraorbital; Cathodal tDCS group: contralesional side; Extra-cephalic tDCS group: contralateral deltoid muscle; Sham tDCS group: Not listed	35 cm^2^	30 s	N/A
Nair et al. (2011)	1	30	5	Contralateral supraorbital area	Contralesional	NA	Stimulation intensity turned off at unspecified interval	Occupational therapy
Hummel et al. (2005)	1	20	2	Ipsilesional	Contralateral supraorbital	25 cm^2^	30 s	N/A

**Table 4:** **rTMS parameters, application location, duration of treatment, and placebo implementation**.

Reference	TMS type	Frequency	Intensity % motor threshold	Number of stimuli per train	Type of placebo or active control	Concomitant Therapy or motor tasks
Fregni et al. (2006)	Contralesional	1 Hz	100%	1200	Sham coil	N/A
Takeuchi et al. (2005)	Contralesional	1 Hz	90&	1500	90°	N/A
Mansur et al. (2005)	Contralesional	1 Hz	100%	600	Sham coil	N/A
Werhahn et al. (2003)	(1, 2, and 4) Contralesional and Ipsilesional (3) Ipsilesional	(1) Single (2) 1 Hz; (3) 1 Hz; (4) single	(1) 130%; (2) 150%; (3) 150%; (4) 130%	(1) *x*; (2) 5; (3) 1800 (4) *x*	Vertex (control stimulation)	N/A
Nowak et al. (2008)	Contralesional	1 Hz	100%	10	Vertex (control stimulation)	N/A
Takeuchi et al. (2008)	Contralesional	1 Hz	90%	1500	90°	N/A
Dafotakis et al. (2008)	Contralesional	1 Hz	100%	600	Vertex (control stimulation)	N/A
Kakuda et al. (2010b)	Contralesional	1 Hz	90%	1200	No sham	Intensive occupational therapy (OT)
Liepert et al. (2007)	Contralesional	1 Hz	90%	1200	Sham coil	N/A
Grefkes et al. (2010)	Contralesional	1 Hz	100%	600	Vertex (control stimulation)	N/A
Lotze et al. (2006)	Contralesional	20 Hz	120%	3	90°	N/A
Lomarev et al. (2007)	Ipsilesional	20 and 25 Hz	110, 120, and 130%	10 and 20	90°	N/A
Malcolm et al. (2007)	Ipsilesional	20 Hz	90%	40	Attaching surface electrodes underneath the magnetic coils and in contact with the scalp connected to the electromyography	Constraint-induced therapy
Khedr et al. (2005)	Ipsilesional	3 Hz	120%	30	Coil angled away from the head	Continued to receive their normal therapy
Khedr et al. (2010)	Ipsilesional	3 and 10 Hz	(1) 130%; (2) 100%	Group (1) 14, Group (2) 20	Coil angled away from the head	combination with conventional therapy
Yozbatiran et al. (2009)	Ipsilesional	20 Hz	90%; and 7 patients 60% device output	40	No sham	N/A
Ameli et al. (2009)	Ipsilesional	10 Hz	80%	50	Vertex (control stimulation)	N/A
Emara et al. (2010)	Contralesional and ipsilesional side	1 and 5 Hz	80–90% ipsilesional 110–120% contralesional	(1) 750 pulses; (2) 150 pulses	90°	received standard physical therapy
Pomeroy et al. (2007)	Ipsilesional	1 Hz	120% MT	40	Sham coil	Voluntary muscle contraction (real and placebo) They were asked to flex and extend the paretic elbow and to continue to repeat or to attempt this for 5 min
Chang et al. (2010)	Ipsilesional	10 Hz	90%	1000	90°	Motor practice consisted of 50 s of reaching and grasping exercises, which were conducted after each rTMS. Plus conventional, physical, and occupational therapy
Kakuda et al. (2011b)	Contralesional	1 Hz	90%	1200	90°	Occupational therapy
Kakuda et al. (2010a)	Contralesional	1 Hz	90%	1200	No sham	Occupational therapy
Koganemaru et al. (2010)	Ipsilesional	5 Hz;	100% of the active motor threshold	(1) 600; (3) 7200	(1) Sham coil; (3) no sham	Exercises for the extensors of the wrist and fingers
Richards et al. (2006)	Ipsilesional	20 Hz	90%	2000	Surface electrodes under the magnet sham rTMS	Constraint-induced movement therapy
Kim et al. (2006)	Ipsilesional	10 Hz	80%	20	90°	N/A
Khedr et al. (2009)	Contralesional and ipsilesional side	(1) 1 Hz; (2) 3 Hz	(1) 100%; (2) 130%	900 pulses	Coil angled away from the head	conventional therapy
Takeuchi et al. (2009)	(1) Contralesional; (2) ipsilesional; (3) bilateral	(1) 1 Hz; (2) 10 Hz; (3) 1 and 10 Hz	90%	(F1) 1000; (F2) 1000; (F3) 2000	90°	N/A
Mally and Dinya (2008)	Contralesional and ipsilesional side	1 Hz	(30% of 2.3 T)	100	No sham	N/A
Kakuda et al. (2011d)	Contralesional	1 Hz	90%	1200	No sham	Intensive occupational therapy (OT)
Kakuda et al. (2012)	Contralesional	1 Hz	90%	1200	No sham	Intensive occupational therapy (OT)
Sasaki et al. (2011)	Contralesional or ipsilesional side	(1) 1 Hz or (2) 10 Hz	90%	1800 or 1000	90°	N/A
Kakuda et al. (2011c)	Contralesional	6 Hz (priming per 10 min) + 1 Hz per 20 min	90%	600 + 1200 = 1800	No sham	Intensive occupational therapy (OT)
Kakuda et al. (2011a)	Contralesional	1 Hz	90%	1200	No sham	Intensive occupational therapy (OT) + Levodopa
Avenanti et al. (2012)	Contralesional	1 Hz	90%	1500 pulses (1 train)	90°	physical therapy
Conforto et al. (2012)	Contralesional	1 Hz	90%	1500 pulses (1 train)	90°	Therapies to outpatient customary rehabilitation,
Chang et al. (2012)	Ipsilesional	10 Hz	80%	50 pulses × 20 trains × 10 daily sessions = 10,000 total	90°	N/A

*Values for each experiment group are separated by bracketed numbers. For example, if an experiment has three groups, the information per group is listed as (1), (2), and (3), respectively.

### Sham: Utilization of placebo stimulation

All the tDCS studies used the same type of sham procedure, which was a brief initial stimulation to produce a tingling sensation followed by decreasing the administration to zero. However, they varied by the duration of initial stimulation, which was 30 or 60 s. The protocols were primarily based on three different strategies: the use of (1) cathodal stimulation in the unaffected hemisphere, (2) anodal in the affected hemisphere, (3) or both anodal and cathodal stimulation applied simultaneously. These three strategies are based on the inter-hemispheric interaction theory described above. The different rTMS parameters, stimulation strategy, and sham type are listed in the Table 4.

Most of the rTMS studies had used sham stimulation or active control stimulation (77.7%), but the techniques used were different; especially in the type of coils and cortical targets used (Table 4). All utilized an rTMS coil but using different approaches: (1) active coil placed on the vertex; (2) active coil, with an angle of application of 90°; (3) sham coil, which induces no magnetic field.

### Failure of improvement: Motor outcomes compared to placebo

A majority of the results was positive for increased improvement compared to placebo, with the exception of three articles (Lomarev et al., 2007; Malcolm et al., 2007; Pomeroy et al., 2007). For the Lomarev et al. (2007) study, results were mixed with some outcomes showing positive results (Lomarev et al., 2007). An important distinction was that the Lomarev et al. (2007) study was primarily implemented to assess safety, while the Pomeroy et al. (2007) study was predominantly designed to test the feasibility of the new methodology (Lomarev et al., 2007; Pomeroy et al., 2007).

Although the article Werhahn et al. (2003) also showed that rTMS induced no improvement or worsening, this study had the main aim of inducing a “Transient, Virtual, Reversible Lesion” to better understanding motor recovery (Werhahn et al., 2003). Another study showing impairment in motor function was the Lotze et al. (2006) study that used rTMS as interference while assessing fMRI data. These results may be secondary to the employment of TMS for inhibition rather than facilitation of motor networks.

### Motor effects size

**Figure 1:**
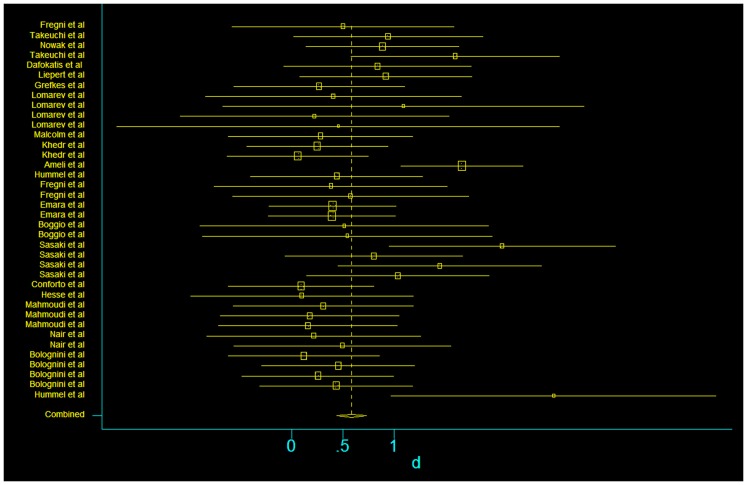
**Forest plot of the subset of studies with amenable and data-available for systematized comparison, with the pooled effect size for studies of transcranial stimulation on motor**.

**Figure 2:**
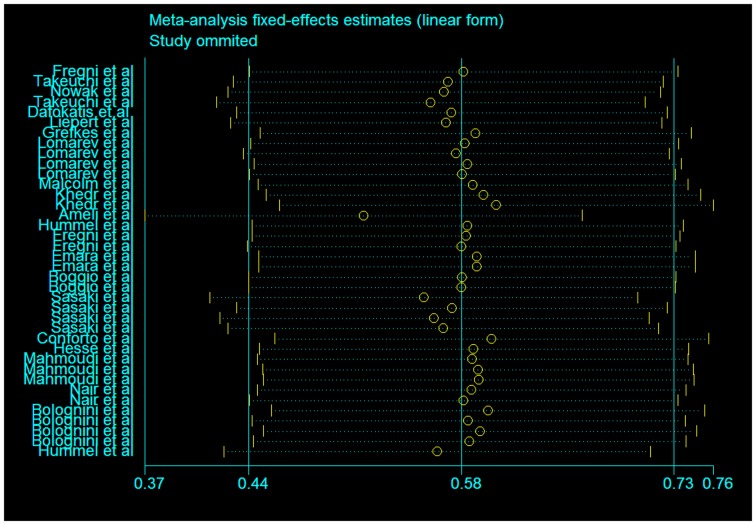
**Assessment of the fixed effects size estimates in linear form with effect size as Cohen’s *d* (standard mean difference) and employing error bars to represent the 95% confidence interval**.

**Figure 3:**
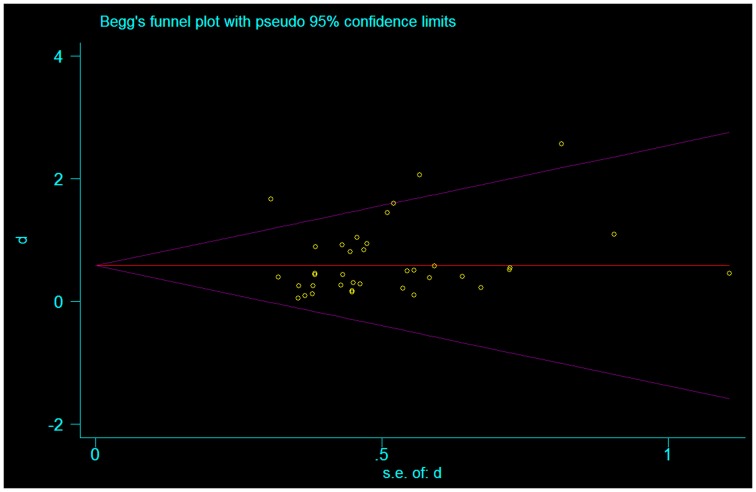
**Funnel plot representing publication bias assessment of the effect sizes (Cohen’s *d*) by accounting for their standard errors**. The pooled effect size is represented by the horizontal solid line. The 94% confidence interval expected for each is represented by the diagonal lines. (Of note, this graph assumes no heterogeneity between studies.)

In our assessment of the magnitude of effect size, we found an overall improvement in motor outcome (Figure 1). Most of the studies used small sample sizes. The results from the fixed effects model revealed a significant pooled effect size of 0.584 (95% CI, 0.440, 0.729; Figures 1 and 2). The random effects model showed similar results 0.590 (pooled effect size, 95% CI, 0.421, 0.760). Using the Begg and the Egger test for the analyzed trials, we found no evidence of publication bias and the distribution of studies was symmetrical with non-significant *p*-values (Figure 3). This suggests that the results are not related to a publication bias. Of note, there were no negative results with tDCS.

### Long-term follow-up

There is a subset of the selected articles that performed long-term follow-up. The time of follow-up varied from 30 min (Takeuchi et al., 2005) to 1 year (Khedr et al., 2010). Khedr et al. (2010) showed a long-term effect lasting 1 year. It is noted in this article that the rTMS was applied in the acute phase of stroke. In the article Yozbatiran et al. (2009), the Fugl-Meyer (FM) did not reveal a difference immediately post-rTMS stimulation, but showed difference 1 week later. In the article Kim et al. (2010b), FM did not demonstrate a difference 1 day after cathode tDCS, but showed a difference 6 months later.

## Discussion

This review of the transcranial stimulation articles includes data from 50 articles, assessing 1314 (1282 stroke patients and 32 healthy) subjects. In summary, the data suggest the use of non-invasive brain stimulation in stroke population is associated with improvements of motor outcomes. There was significant heterogeneity of patient population characteristics, intervention parameters, and selected assessments.

### Studies retrieval

Though the yearly number of studies did not vary significantly, there was an overall increase in publications over time (years) that peaked in 2011. The publications averaged at 4.9 articles per year. In order to attain a larger perspective, we compared this trend with a trajectory of the overall trend of non-invasive articles publications. The comparative trend was obtained from a PubMed search utilizing the search terms of “stroke” and “transcranial stimulation” until the publication year of 2011. Of note, the comparison trend used data searched until the end of December of 2011 in order to provide a clear trend for the whole year of 2011. When assessing for tDCS alone (utilizing the same search terms and “tDCS” or “direct”), the data also demonstrated an increase in publications from its 0 to 2 yearly publication rate to recently 47 articles for 2011 (a 235% increase from PubMed publications of 2002). Lum et al. (2002) reports that the increased drive for novel therapies in stroke rehabilitation is indirectly actuated by an emerging cost-reduction emphasis in healthcare. Other articles also support this hypothesis by proposing a socioeconomic justification for the search for new stroke therapies (Edwards and Fregni, 2008; Nowak et al., 2009). The increasing popularity of novel therapies is suspected to be due to the sustained impact of chronic disability in stroke (Lum et al., 2002; Edwards and Fregni, 2008; Nowak et al., 2008). This observation is supported across the literature, as other sources have noticed that both tDCS and rTMS are experiencing an emerging popularity of use in the field of medicine and research (Ryan et al., 2006; Harris et al., 2008; Funke and Benali, 2011; Schwarzkopf et al., 2011; Fox et al., 2012; Hellmann et al., 2012). Ratan and Noble (2009) argues for the need for infrastructural support to facilitate development and translation of novel therapies. Kent et al. (2009) also suggests advocacy for use of advanced technology to develop models between neuroplasticity and learning in stroke recovery. In summary, the field of medical research suggests that the field of non-invasive stimulation is an emerging field with a potential role in stroke rehabilitation.

### Effects of age on brain stimulation and stroke

In regards to age, the average age of this systematic review was 58.46, which is a low average when compared to the general stroke population. A comparison to the other reviews of stroke in the literature reveals a meta-analysis of therapy and stroke that reports older patient averages to be 65.3–74.7 years for their respective treatment groups (Craig et al., 2010). Investigation of the study design of our selected articles demonstrates that this finding is not attributed to the inclusion/exclusion criteria or adverse events (Table S1 in Supplementary Material). Of note, a recent review of non-invasive stimulation established an average patient age of 58.77 (Richards et al., 2008), a report similar our age finding.

Given that certain articles have discussed safety concerns with extremities of age, we considered whether the average age was related to safety concerns (Quintana, 2005). Since age did not correlate with safety reports in these articles. A potential explanation is an increased utilization of new treatments in the younger stroke population (Luker et al., 2011). This trend is substantiated by a recent review of stroke management, where age is evidenced to be a significant determinant of type of post-stroke care (Luker et al., 2011). Furthermore, according to the TMS guidelines, age does not increase the risk of adverse events in the utilization of TMS.

We analyzed the relationship between effect-size of motor outcomes after non-invasive stimulation with age. We noticed no correlation (*r* = 0.279, *p* = 0.0984) between age and effects size when using a linear regression model. The Pearson coefficient was very low and the *p*-value was high, which conveys a poor association and low significance. A comparison of effect sizes of patients above and below the median age (55.9) also failed to reveal a significant difference in age groups and motor outcomes (Mann–Whitney *U*-test: *p*-value 0.101694, two-tailed test.) Sub-analysis of age by rTMS and tDCS articles also failed to show a significant difference (Mann–Whitney two-tailed *U*-tests: rTMS *p* = 0.1246498, tDCS *p* = 1). We conclude that our analysis was unable to find a difference or association in effect sizes of motor outcomes when analyzed by age.

Future studies would be helpful in further exploring this concept of age and motor outcomes in transcranial stimulation. Literature suggests that there exists an increased level of neuroplasticity in younger population (Pinto et al., 2012). This may be an important consideration in transcranial stimulation of stroke patients to determine if younger patients would experience increased motor improvements. Some data suggests that younger patients may experience greater improvement based upon an increased ability of the contralateral hemisphere to compensate for the stroke lesion (Ipek et al., 2011). Studies should explore whether the level of cerebral atrophy in the setting of older age should be a consideration for analyzing age-related motor effects (Nahas et al., 2004; Decarli et al., 2012). Further studies are needed to explore fully the relationship between age and motor outcome after transcranial stimulation in stroke patients.

### Effects of gender on brain stimulation and stroke population

The analysis failed to find significant correlation; however there was a slightly positive trend for increased effect size as male percentage increased and a conversely decreased correlation of effect size where the percentage of females were higher. In comparison with the literature, a study on chronic tinnitus with tDCS demonstrated an opposite trend with females improving more than males (Frank et al., 2012). Another study in tDCS on behavior modification and reasoning also found an increased effect in women (Fumagalli et al., 2010). Furthermore, a study of temporal cortex tDCS on its effects on facial expression recognition also noticed increased effects and modulation of the cortex with females (Boggio et al., 2008).

Overall, the findings of this review did not provide sufficient information to draw definitive conclusions on the effects of gender. Results may be related to statistical sampling and analysis. An explanation for why the results failed to find increased effects with female-predominant articles is that the findings are masked by the uniqueness of stroke epidemiology compared to the other diseases studied in other articles. As described above, the average patient age of this study was 57. According to the AHA, men tend to have more strokes at an earlier age than women do (Lloyd-Jones et al., 2009). Ergo, one would expect fewer females in our articles. This decreased number of females may be relatively too few (in comparison to the male patients) to demonstrate a preferential improvement in motor outcome. This epidemiological trend of more males than females is supported in this review’s high male:female ratio of 63:37%. The variability in number of male versus female patients in these articles may also be due to varying recruitment or level of desire/comfort with neuromodulation treatment. Once again, it should also be emphasized that there are no contraindications against non-invasive stimulation for either gender (Rossi et al., 2009). Further studies should assist in delineating this effects of stimulation in gender, as some articles report there is a differential effect (Knops et al., 2006; Boggio et al., 2008; Chaieb et al., 2008). This information may prove paramount in helping to individualize stimulation treatment.

### The impact of the chronicity of stroke on the recovery of motor function

As listed above, there is significant variability in the phase of stroke for which the patient received the stimulation between the articles. This variability of time after stroke also exists between subjects within the articles. This variability may be more meaningful when it is related with acute/subacute stroke than the ones related to chronic stroke. One specific issue we noticed in the articles is that overall, there was no consensus as to what was considered acute, subacute, or chronic stroke. We further discuss some solutions below.

The articles that applied stimulation in patients within approximately 1 month or less after stroke demonstrate significant heterogeneity in post-stroke duration, both intra and inter-study: Liepert et al. (2007), 7.3 days (SD: 4.5); Khedr et al. (2010), 6.5 days (SD: 3.63); Khedr et al. (2005), 7.1 ± 1.4 days for active stimulation and 7.3 ± 1.5 days for sham; Chang et al. (2010), 13.4 days with range 7–26 (12.9 ± 5.2 days for active stimulation, 14.4 ± 5.9 days for sham); Khedr et al. (2009), 17.1 days (SD: 3.6); (Kim et al., 2010b), 34.0 ± 27.1 for anodal tDCS, 19.4 ± 9.3 for cathodal tDCS, and 22.9 ± 7.5 for sham; Sasaki et al. (2011), 18.4 ± 5.8 days for high-frequency rTMS, 17.0 ± 6.0 days for low frequency rTMS, 15.4 ± 4.3 days for sham; Hesse et al. (2011), 23.8 ± 12.6 for anodal tDCS, 26.6 ± 9.8 for cathodal stimulation, and 26.6 ± 10.5 days for sham; Conforto et al. (2012), 27 ± 8.6 days for active stimulation and 28.3 ± 10.5 days for sham stimulation (Khedr et al., 2005, 2009, 2010; Liepert et al., 2007; Chang et al., 2010; Kim et al., 2010b; Hesse et al., 2011; Sasaki et al., 2011; Conforto et al., 2012).

This may further serve as a confounding factor, as the responses may differ with this variance. In these articles, the stimulation paradigms were employed with approaches based upon the inter-hemispheric theory. However, there are other mechanisms of neuronal recovery that may be applicable and worth consideration.

For instance, it is suspected that the NMDA receptor may play an important role in acute phase, in preventing neuronal death in the penumbra area. There is a theory that postulates a possible bipartite capacity of NMDA receptor after the stroke: (1) it is possible that in the early stage after stroke the overactivation of NMDA seems to be detrimental; (2) on the other hand, in a delayed phase this activation may be essential for neuronal recovery (Lo, 2008).

Since, tDCS and TMS seems to have effects on the NMDA receptors (Kim et al., 2010a), further studies are necessary to define the best moment to alter NMDA activity after stroke. Studies may then use this data to decide the best application for these neuromodulatory techniques. It is possible that the best approach is to use low frequency rTMS and cathode tDCS in the hyperacute/early phase and high-frequency rTMS and anodal tDCS in the chronic/later phases.

This suggestion is in light of a theory that rTMS may increase brain metabolism (Valero-Cabre et al., 2007), which may be harmful for the penumbra area. On the other hand, there is evidence that rTMS may decrease apoptosis after stroke (Gao et al., 2010). Gao et al. (2010) has shown that high-frequency rTMS therapy increased glucose metabolism and inhibited apoptosis in the ischemic hemisphere of a rat model of transient cerebral ischemia. Similarly, Yoon et al. (2011) has demonstrated a role of diminishing apoptosis in the 20 cerebral ischemic rats after a 10-Hz frequency were applied to the ipsilesional cortex at day 4 after cerebral ischemia. Considering this evidence, a conservative approach would be to opt for low frequency rTMS in contralateral hemisphere like Liepert et al. (2007) since it circumvents increasing brain metabolism by avoiding direct action on the penumbra area. These parameters may provide a more protective effect.

The question that remains is how such an intervention will alter the trajectory of the stroke over time. It appears that this can alter the natural recovery of the stroke, as evidenced by Khedr et al.’s (2010) improvement at 1-year follow-up.

The lack of consensus in definition of acute versus chronic phases of stroke is one of the main issues in post-stroke duration. Without a standardization of this description, analysis, and generalizations of implications are going to be limited in the future. Perhaps maintenance of a stringent classification system would facilitate studying the safety and other effects of stimulation, as well as the time course of neuroplasticity. Although none of the articles demonstrated worsening motor function during the acute phase, the question remains whether this is safe to perform during the acute phase. There were not enough acute articles included in the effect size analysis to obtain a difference in the acute versus chronic stage. However, we continue to raise the question of whether (and how) the strategy during acute phase should differ. Overall, we contemplate as to whether implementation of neuromodulation during the acute period will block maladaptive plasticity. Perhaps it would also enhance beneficial plasticity and early recovery. Yoon et al. (2011) article supports this use by demonstrating the role of diminishing apoptosis in the post-stroke period.

In this regard, we suggest using the definition of Bahn et al. (1996) of stroke stages: hyperacute: the first six post-ictal hours; acute: 6–24 h; subacute: 24 h to 6 weeks; chronic: greater than 6 weeks. By this classification, this would make all the selected articles, subacute and chronic. Perhaps employment of this system will help with standardization. Overall, we anticipate that this will be an exciting area of research and development in the future.

### Effect of the magnitude and nature of the stroke on motor outcome: Stroke severity and localization

There was significant heterogeneity in the severity of strokes reported in the articles. Table 1 shows the severity of strokes listed. We question whether the severity of stroke provides better or worse potential for neuroplasticity, or if this issue confounded by the ability to measure response. There are stroke articles that demonstrate significant improvement even with markedly severe strokes, which helps illustrate that the mechanism is effective. Specifically, we refer the reader to a case report that evinces post-stimulation improvement of a severe stroke subject (Boggio et al., 2006). This aspect requires further delineation in the future studies by standardization of the level of stroke in study participants.

The trend of heterogeneity of study population continues in the localization of the stroke. This is also delineated in Table 1. Out of the articles analyzed for effect size, there were eight results that studied subcortical strokes, while the remaining effect sizes were articles using both cortical and subcortical stroke. The analysis demonstrated a highly significant increased effect size when stimulation was applied to subcortical strokes versus the mixed strokes (*p* = 2.45598e−05, two-tailed Mann–Whitney *U*-test.) When sub-analyzed within the context of type of stimulation technique, this significant finding was reproducible for both rTMS and tDCS articles (rTMS *p* = 0.01115294 and tDCS *p* = 0.01428572, Mann–Whitney two-tailed *U*-test). The increased effect size in articles with subcortical strokes leads to an interesting point. As described in the introduction, one of the primary observations that made non-invasive stimulation of stroke patients worthy of discussion was based on changes in cortical excitability. In essence, the neuroimaging findings of cortical excitability and other descriptions of inter-hemispheric inhibition (Hummel and Cohen, 2006) are all observations that occur in the neuronal cortex. Therefore, it is possible that the subcortical strokes preserve the cortex and allow neuroplasticity and neuroadaptation of the post-stroke maladaptive changes. This explanation may be the main component underlying the improvement in the subcortical patients. In corroboration, it is notable that one of the selected articles also supports this finding, by describing greater improvement with subcortical versus cortical stroke (Ameli et al., 2009). If this is a re-demonstrable finding, then it may be possible to utilize stroke localization in the future as a means of treatment stratification and perhaps even a predictor of response.

### Adjuvant therapy

The adjuvant therapy results are listed in Tables 3 and 4. There was insufficient data to analyze the type, order, and effect of adjuvant therapy on motor effect size. We contemplate whether the sequence of stimulation and adjuvant therapy interfered with the results. Specifically, does implementing therapy pre-, post-, or co-stimulation affect the overall motor effect? Perhaps therapy provides a priming effect, or conversely interrupts the neuromodulatory learning. An interesting point of consideration especially with negative studies is whether the adjuvant therapy is the limiting factor influencing the observed results. Is there an underlying type II error present? It may be that there is a ceiling effect on motor improvement achievable after stroke for some patients. In those cases, it may be that the therapy increases the outcomes to the ceiling, thereby making it impossible to detect any further improvement that would have been attained from the stimulation application. There are few articles that compare constraint induced therapy and rTMS (Richards et al., 2006; Malcolm et al., 2007) but were unable to establish a difference. However, with tDCS, it has been demonstrated that tDCS has an additional benefit when applied on top of constraint induced therapy in healthy (Williams et al., 2010) and stroke subjects (Bolognini et al., 2011).

### Adverse effects and safety considerations in the implementation of neuromodulation

These articles highlight certain concerns previously raised regarding the safety parameters of rTMS. Recent articles advocate for higher doses of rTMS application in order to optimally define the most efficacious paradigm (Hadley et al., 2011). Current safety protocols that guide treatment are based on ascertaining the spread of cortical activity after stimulation in healthy patients (Pascual-Leone et al., 1993; Rossi et al., 2009). Studies such as Benninger et al. (2009) have shown doses as high as 50 Hz have been safely administered in the Parkinson’s population. However, it is imperative to determine how this spread of cortical activity will be altered in stroke patients.

A cardinal reason that dose optimization must occur in the stroke population is the potential for epileptogenic events (Burn et al., 1997; Rossi et al., 2009) According to Olsen (2001), compared to the general population, the risk of developing seizures is 35 times more likely in the stroke population in the first year after stroke and 19 times more likely in the second year after stroke. Another study documents the risk of seizure as 23 times more likely the first year of stroke and remained increased over the following three post-stroke years (So et al., 1996). Out of the selected articles, one article noted a spread of electromyographic activity, denoting a possible peripheral manifestation of cortical-excitation spread, as per the suggestion of the Pascual-Leone et al. (1993) article (Pascual-Leone et al., 1993; Lomarev et al., 2007). Lomarev et al. (2007) further suggests the safety parameters may be different for healthy and stroke subjects. Therefore, for patients with additional risk, rigorous monitoring is still critical (Rossi et al., 2009).

Given the aforementioned guidelines of cortical activity were initially based on healthy subjects, it is still to be determined the exact dose that will elicit a spread of cortical activity in stroke patients. Specifically, it will be imperative to determine the stimulation parameters and the stroke subtype characteristics for which they are applicable. For example, there exists concern that the ischemic region of the stroke might be more epileptogenic than healthy tissue, thereby increasing the need for vigilance during stimulation. Although the overall cause of epileptic seizures is poorly understood, Olsen (2001) offers that the substrate of the seizure is likely attributed to the ischemic penumbra surrounding the stroke lesion. The enhanced release of excitotoxic glutamate, disintegration of membrane material, ionic disruption, and release of inter-neuronal substance is also implicated (Olsen, 2001). Hemorrhagic stroke subtypes are described as being more epileptogenic (Kilpatrick et al., 1990; Reith et al., 1997). In the 1997 Copenhagen Stroke Study, post-stroke seizures were found to be more common in the hemorrhagic group than the ischemic stroke group (Reith et al., 1997). Furthermore, Bladin et al. (2000) also describes how stroke type (ischemic versus hemorrhagic) impart different seizure risk. Burneo et al. (2010) also lists stroke severity and presence of hemorrhage as risk factors for seizure after stroke by multivariate analysis. Within our selected article group, there are articles that consider this caution by excluding patients with hemorrhage due to suspected increased risk of seizure (Carey et al., 2008). It is worth consideration that the presence of hemorrhage may require specific safety recommendations in the future.

An additional consideration is that some studies note that hemorrhagic stroke occurred exclusively in patients with cortical involvement of the stroke territory (Kilpatrick et al., 1990). This may imply a safety rationale for different stimulation protocols for cortical versus subcortical strokes.

These above considerations make it evident that further consideration and a more in-depth discussion of the stroke characteristics may be warranted for tailoring and development of future stimulation protocol. Furthermore, as the doses of high-frequency rTMS are advanced in the future, acquisition of studies as performed in the stroke population will be warranted to establish supporting safety data (Lomarev et al., 2007). In the interim, many options using modalities of low and moderate frequency rTMS exist to explore their role on neurorecovery of motor function.

One should note however that although seizure events are highly discussed, there have been none reported in this current literature group. It should also be noted historically that the seizure events that have been reported in TMS history have been frequently associated with secondary causes such as medications, past medical history, environmental factors, outside of the TMS alone. In fact, according to recent rTMS guidelines by Lefaucheur et al. (2011), most of the reports in the literature were secondary to parameters that did not previous recommendations or concomitant use of medications that lowered the seizure threshold. However, for the sake of prudence, seizures should continue to be kept high on the differential of concerns when discussing safety. Though the use of psychotropic medications has been reported to increase the risk of seizure (Rossi et al., 2009), it is undetermined how many of these medication warrant exclusion as an official exclusion criteria. Table S1 in Supplementary Material indicates that only few articles considered these criteria. Furthermore, some of these articles contend that the exclusion of these medications was not for safety reasons but rather for quality outcomes, to avoid medication interference with the results.

Other safety concerns peri-stimulation include changes in cognition and mood. Although this adverse event was not reported in the selected articles, it is worth noting that most of them did not measure for them. This is an interesting omission considering the FDA approval for rTMS use for is for depression which is a mood disorder (Dell’osso et al., 2011). Consequently, this alone should provide sufficient incentive to include this category in the safety outcomes in stimulation studies. Specifically, it would be beneficial to ascertain how stroke location, severity, and choice of stimulation parameters affect the outcomes. Future studies would be helpful in discriminating these issues.

Further studies should also explore the ideal safety-monitoring device, whether it be EEG or development of specific biomarkers. Before implementation, one should considering referencing multiple sources of safety reviews of tDCS and TMS treatments (Rachid and Bertschy, 2006). Certain articles compare TMS field distributions for healthy versus stroke tissue (or atrophy or tumor), noticed modified current density distributions and alterations for stimulation proximal to the stroke (Mansur et al., 2005; Wagner et al., 2008). Safety guidelines also suggest that further EEG studies are needed to collect data on various parameters on stimulations (Rossi et al., 2009). Overall, further studies analyzing the effects of protocols using high-frequency rTMS would be helpful in determining specialized safety parameters in order to individualize recommendations for high versus low frequency rTMS.

### Stimulation protocol and parameters

There were multiple variations of parameters employed in the selected studies, mostly to improve inter-hemispheric imbalance (with the exception of Mally and Dinya, 2008). Mally and Dinya (2008) given that some of the articles selected motor as a secondary (rather than primary) outcome, it is possible that they selected parameters that were more efficiently measured in a single session of stimulation. In comparison, certain articles such as Fregni et al. (2006) report that repeated sessions are helpful in maintaining efficacy. Therefore, one can expect some heterogeneity in the implications of the results attained.

The concept of inter-hemispheric interaction and balance is further considered in most of the selected studies (as described above). This hypothesis is a prevailing theory in the field and is reinforced by certain studies such as Werhahn et al. (2003). However, we contemplate in our review whether the contra-lesional hemispheric hyperactivity denotes an additional purpose. Specifically, Lotze et al.’s (2006) investigation demonstrates where stimulation of contra-lesional hemisphere can contribute to further impairment of the paretic hand. It is to be considered that the hyperactivity in the contra-lesional hemisphere may be beneficial in a small particular subset of strokes. This may be specific for subjects with complete motor recovery. The implication may be that stimulation protocols should be individualized to the level of recovery, especially in this subset of stroke patients.

The second consideration regarding the inter-hemispheric interaction theory is the role of the healthy hemisphere. There are some articles that propose that the healthy hemisphere can play a role in the recovery of stroke in the subset of patients who have experienced improved recovery. Perhaps the stimulation protocols should also take account of this population of patients.

In practicum, it is important to be able to translate these effects and principles of inter-hemispheric interaction to the generalized stroke population. Specifically, this analysis raises the point of how stimulation should be altered in the setting of bilateral strokes. In this patient population, would the same alteration of inter-hemispheric balance still be applicable? If so, does one select the side to inhibit or facilitate based on the severity of the contralateral side? Would the improvement in one side be at the expense of the other side’s motor or cognitive effects? What excitability relationship does the new and the previous lesion have with one another in the balance of inter-hemispheric interaction? How do we propose to balance their effects? These questions are not only applicable in the understanding of the inter-hemispheric interaction, but also its application. It becomes especially tangible given the high risk of yearly recurrence of stroke of 185,000 in the United States alone. The scenario of multiple strokes is significantly common. Ergo, this topic would benefit from further consideration in the design and optimization of this intervention.

The concept of intra-cortical facilitation should also be further optimized. As has been previously demonstrated, the activity of the peri-lesional region can be increased with non-invasive stimulation (Takeuchi et al., 2009). However, attempts should further be made to delineate the localization of application. How does one definitely determine the ideal location? Should it be by fMRI, EEG, or optimal scalp position (OSP)? If so, how does one compensate with tDCS paradigms, given there is a difference between the electrode placement and the exact location where the current is flowing. In order to direct treatment, one would have to provide accurate parameters and titration guidelines in order to provide prescriptions that will effectuate improvement in care.

It is undetermined if the stroke recovery to the primary motor cortex has a specific role in improvement of dexterity (Rouiller et al., 1998). In certain articles, there is an improvement in dexterity without improvement in force. In these cases, is the improvement in dexterity due to a particular predilection for dexterity in the motor cortex? Alternatively, is there a relative higher difficulty in improvement of force generation in the lesioned patient? (Sohn et al., 2002; Liepert et al., 2007). Elucidation of this aspect will also help to individualize stimulation parameters and select motor assessment outcomes.

### Sham: Utilization of placebo stimulation

Given the earlier discussion on the unknown optimal protocol for stimulation, one may question whether one is inducing motor changes in the 90° and vertex sham stimulation methods. This might be even more applicable in patients with stroke who have or are currently undergoing neuroplasticity of cortical pathways. It is unclear if there are effects on these new or old motor pathways in producing an alteration in motor outcome. One would need to determine how well these procedures mimic active (or real) stimulation without producing confounding changes in order to provide a more ideal unblemished placebo comparison.

The issue of placebo is an aspect that will need to be addressed in future studies. As mentioned above, not all the articles included in our review used a placebo group to compare against the intervention groups. There was also a significant amount of heterogeneity in the type of placebo. Therefore, a standardized sham stimulation protocol must be initiated in order to rule out placebo effects out in non-invasive brain stimulation intervention studies. This is especially important in the context that motivation to perform an activity may be associated with a noticeable placebo effect. Considering that these therapies involve constant contact with researchers or therapists, it may present some positive effects over the patients’ rehabilitative drive and motor effort. Because these studies did not sufficiently sham or mask treatment, it is possible that the results found were due to a placebo effect. However, since there were also improved measures of cortical excitability, it is less likely the improvements observed were related to increased effort alone. Nevertheless, randomized sham-controlled trials that explore non-invasive stimulation would have to be a standard in the future development of non-invasive brain stimulation studies in the stroke population.

### Motor assessment tools and effects: Strength versus dexterity

Given the numerous different assessment tools that were utilized for the neurostimulation articles, we simplified them into Table 2. They varied in the types of assessment tools and their times of implementation. These assessment tools have the ability to study different aspects of motor function and impairment. This point of cogitation generates a discussion whether an outcome is an optimal assessment. Given the broad concept of motor ability, each motor movement is comprised of multiple different sub-abilities that involve various parts of the brain and neurological system. We contemplated the optimal state of the measures being studied whether one is studying clinical, research, or surrogate outcomes. An Australian study explores this point in stroke survivors by noting that inclusion of consumers to gage and rank personal significance and implications of motor outcomes can be helpful in research priority setting (Sangvatanakul et al., 2010). Park et al. (2008) establishes that baseline clinical measurements and research motor assessments can be used to predict clinically meaningful outcomes in patients with stroke (Park et al., 2008). This may be an interesting colloquy in the future, as the determination of improvement is translated to the clinical arena.

We explore the concept of motor implications in our discussion of strength versus dexterity. In the selected articles, there were articles that did not show increase in strength, but showed increased dexterity instead (Liepert et al., 2007). This phenomenon of dissociation of strength and dexterity is well described by Noskin et al. (2008), who studied 30 patients with first time unilateral strokes. They hypothesized that the ipsilateral hand could be proven to be functionally impaired from the initial 24–48 h of the stroke and up to 1 year of follow-up (Noskin et al., 2008). They successfully predicted that the impairments of dexterity and strength would diverge both in the acute phase and in the recovery process, with the aim of proving independent modes of malfunction (Noskin et al., 2008). As further evidence, the impairments in dexterity maintained correlations with one another despite the lack of correlation between dexterity and stroke impairments (Noskin et al., 2008).

This raises the following questions: (1) whether it is easier to provide improvement in dexterity versus strength; (2) whether it requires more neuronal improvement/preservation to generate more force than dexterity; and (3) whether dexterity improves based on recruitment of additional neuronal tracts versus recovery of the original impaired neurons. Noskin et al., 2008 suggests that the various aspects of motor function require multifarious degrees of bilateral cortical involvement and input (Noskin et al., 2008). This is supported by fMRI data that demonstrate that various complex motor tasks require bihemispheric activity, especially for motor planning, sequencing, and integration of sensorimotor information (Haslinger et al., 2002; Filippi et al., 2004; Krakauer, 2005; Poldrack et al., 2005). Furthermore, TMS data has proffered accepted elucidations of ipsilateral impairments through the concept of inter-hemispheric interactions via transcallosal connections (Haaland and Delaney, 1981; Haaland and Harrington, 1989; Shimizu et al., 2002). This hints that the concept of dexterity is a multi-faceted sub-component of motor function that likely differing effects and outcomes from the stroke. The literature also suggests that the post-stroke motor network is influenced by other neuronal phenomena such as deafferentation, and circuit connections with the basal ganglia and cerebellum (Parent and Hazrati, 1995; Schmahmann and Pandya, 1997). A simpler question is whether it is more difficult to modify force generation (Rouiller et al., 1998; Sohn et al., 2002; Liepert et al., 2007)? Recent literature demonstrates that it is possible to apply non-invasive stimulation (cathodal tDCS) to the cerebellum to invoke motor adaptive learning improvement (Galea et al., 2011). We question if the dissociation between strength versus dexterity improvement would be further elucidated with stimulation was applied to the cerebellum instead of the motor cortex. These studies adumbrate the point of the variability of outcome assessments used in the articles. It is capital that future studies design specifically for strength and dexterity outcomes and localize these changes to the motor cortex or the respective involved loci. The application of the above aspects and sub-classification of motor function would be informative and essential for future studies to evaluate the comparisons of learning and improvement in neuroplasticity.

### Long-term

As described above, there is a subset of the selected articles that performed long-term follow-up. The time-periods varied from 30 min (Takeuchi et al., 2005) to 1 year (Khedr et al., 2010). A distinctive observation of the Khedr et al. (2010) article is the prolonged duration of preserved effects. An aspect that makes it to be particularly informative is that the stimulation was implemented in the acute phase of the stroke. This highlights the earlier discussion on the ideal window of time for intervention, whether it is beneficial to intervene early or later in the course. Due to this study, we contemplate if chronic stroke cases would show further improvement if follow-up was provided greater than 1 year. Another notable observation was regarding the type of assessment used for follow-up. It is worthy of discussion that the Fugl-Meyer score did not reveal an improvement immediately post-rTMS stimulation in another study but showed a difference 1 week later (Yozbatiran et al., 2009). It may that the long-term improvements that occur after stimulation are due to long-term potentiating effects and therefore manifest slowly and gradually over time. This might explain the trend of delayed improvement noted in the Fugl-Meyer (Yozbatiran et al., 2009).

### Limitations

There are some limitations related principally to the information content in the selected article. Some articles did not provide necessary information to calculate the effect size, besides they did not give enough demographic information of the patient and better description of side effect.

### Recruitment

One problem with study recruitment is that it is that novel therapies are typically only available in academic areas. Thereby, the study groups would be primarily comprised of patients who reside in proximity to these areas. This may limit the generalizability of results in non-academic populations. Moreover, these patients may have differing access to acute stroke management, given the narrow window of antithrombotic treatment. This may also affect the generalizability to rural and lower-access regions. It may be interesting in the future to appraise how increases in the availability of these therapies affect the epidemiological outcome and translational applicability.

### Future directions

This review shows that there is a plethora of areas that need to be studied in the field of neuromodulation to optimize the analysis of motor recovery of stroke patients. Although many of the future suggestions were listed above, we summarize some of them here. In essence, future directions would lead toward the standardization of investigation and application. Specifically, future studies will have to evaluate motor assessments and elucidate which would be the most prudent and applicable choice. We will have to further evaluate safety parameters of stroke patients, especially as we explore the future use of high-frequency rTMS. Future studies should help develop homogeneity in sham procedures as well. Most importantly, it would be helpful to ascertain motor assessment data that are attuned to specific stroke baseline characteristics (age, stroke duration, and stroke location), which would facilitate individualization and optimization of treatment.

## Conclusion

From this analysis of collected studies (Tables 1–4), it is observable from the available data that non-invasive stimulation may beneficial in enhancing motor recovery. Specifically, it may lead to clinically meaningful functional motor improvements in the stroke population. Future studies would benefit from future standardization of outcomes and stimulation parameters in order to decrease variability and heterogeneity of results. Future studies should also help delineate the subtypes of patients that do not benefit from specific parameters. These changes would be helpful in understanding how to individualize therapy to various stroke sub-populations, with the aim of the optimization of neurorecovery of motor function.

## Conflict of Interest Statement

The authors declare that the research was conducted in the absence of any commercial or financial relationships that could be construed as a potential conflict of interest.

## Supplementary Material

The Supplementary Material for this article can be found online at http://www.frontiersin.org/Neuropsychiatric_Imaging_and_Stimulation/10.3389/fpsyt.2012.00088/abstract

Supplementary Table S1 **Adverse events and safety outcome for selected peer-reviewed articles**. MMSE, mini-mental state examination; EEG, electroencephalography.

## References

[B1] Ameli M. Grefkes C. Kemper F. Riegg F. P. Rehme A. K. Karbe H. (2009). Differential effects of high-frequency repetitive transcranial magnetic stimulation over ipsilesional primary motor cortex in cortical and subcortical middle cerebral artery stroke. Ann. Neurol. 66, 298–309 10.1002/ana.21725 19798637

[B2] Avenanti A. Coccia M. Ladavas E. Provinciali L. Ceravolo M. G. (2012). Low-frequency rTMS promotes use-dependent motor plasticity in chronic stroke: a randomized trial. Neurology 78, 256–264 10.1212/WNL.0b013e3182436558 22238412

[B3] Bahn M. M. Oser A. B. Cross D. T. III. (1996). CT and MRI of stroke. J. Magn. Reson. Imaging 6, 833–845 10.1002/jmri.1880060518 8890023

[B4] Benninger D. H. Lomarev M. Wassermann E. M. Lopez G. Houdayer E. Fasano R. E. (2009). Safety study of 50 Hz repetitive transcranial magnetic stimulation in patients with Parkinson’s disease. Clin. Neurophysiol. 120, 809–815 10.1016/j.clinph.2009.01.012 19285918 PMC2667907

[B5] Bladin C. F. Alexandrov A. V. Bellavance A. Bornstein N. Chambers B. Cote R. (2000). Seizures after stroke: a prospective multicenter study. Arch. Neurol. 57, 1617–1622 10.1001/archneur.57.11.1617 11074794

[B6] Boggio P. S. Alonso-Alonso M. Mansur C. G. Rigonatti S. P. Schlaug G. Pascual-Leone A. (2006). Hand function improvement with low-frequency repetitive transcranial magnetic stimulation of the unaffected hemisphere in a severe case of stroke. Am. J. Phys. Med. Rehabil. 85, 927–930 10.1097/01.phm.0000242635.88129.38 17079967

[B7] Boggio P. S. Nunes A. Rigonatti S. P. Nitsche M. A. Pascual-Leone A. Fregni F. (2007). Repeated sessions of noninvasive brain DC stimulation is associated with motor function improvement in stroke patients. Restor. Neurol. Neurosci. 25, 123–129 17726271

[B8] Boggio P. S. Rocha R. R. da Silva M. T. Fregni F. (2008). Differential modulatory effects of transcranial direct current stimulation on a facial expression go-no-go task in males and females. Neurosci. Lett. 447, 101–105 10.1016/j.neulet.2008.10.009 18926878

[B9] Bolognini N. Vallar G. Casati C. Latif L. A. El-Nazer R. Williams J. (2011). Neurophysiological and behavioral effects of tDCS combined with constraint-induced movement therapy in poststroke patients. Neurorehabil. Neural Repair 25, 819–829 10.1177/1545968311411056 21803933

[B10] Burn J. Dennis M. Bamford J. Sandercock P. Wade D. Warlow C. (1997). Epileptic seizures after a first stroke: the Oxfordshire Community Stroke Project. BMJ 315, 1582–1587 10.1136/bmj.315.7122.1582 9437276 PMC2127973

[B11] Burneo J. G. Fang J. Saposnik G. (2010). Impact of seizures on morbidity and mortality after stroke: a Canadian multi-centre cohort study. Eur. J. Neurol. 17, 52–58 10.1111/j.1468-1331.2009.02739.x 19686350

[B12] Calautti C. Baron J. C. (2003). Functional neuroimaging studies of motor recovery after stroke in adults: a review. Stroke 34, 1553–1566 10.1161/01.STR.0000071761.36075.A6 12738893

[B13] Carey J. R. Evans C. D. Anderson D. C. Bhatt E. Nagpal A. Kimberley T. J. (2008). Safety of 6-Hz primed low-frequency rTMS in stroke. Neurorehabil. Neural Repair 22, 185–192 17876070 10.1177/1545968307305458

[B14] Chaieb L. Antal A. Paulus W. (2008). Gender-specific modulation of short-term neuroplasticity in the visual cortex induced by transcranial direct current stimulation. Vis. Neurosci. 25, 77–81 10.1017/S0952523808080097 18282312

[B15] Chang W. H. Kim Y. H. Bang O. Y. Kim S. T. Park Y. H. Lee P. K. (2010). Long-term effects of rTMS on motor recovery in patients after subacute stroke. J. Rehabil. Med. 42, 758–764 10.2340/16501977-0637 20809058

[B16] Chang W. H. Kim Y. H. Yoo W. K. Goo K. H. Park C. H. Kim S. T. (2012). rTMS with motor training modulates cortico-basal ganglia-thalamocortical circuits in stroke patients. Restor. Neurol. Neurosci. 30, 179–189 22555430 10.3233/RNN-2012-110162 PMC3589123

[B17] Conforto A. B. Anjos S. M. Saposnik G. Mello E. A. Nagaya E. M. Santos W. Jr. (2012). Transcranial magnetic stimulation in mild to severe hemiparesis early after stroke: a proof of principle and novel approach to improve motor function. J. Neurol. 259, 1399–1405 10.1007/s00415-011-6364-7 22173953 PMC4883097

[B18] Craig L. E. Bernhardt J. Langhorne P. Wu O. (2010). Early mobilization after stroke: an example of an individual patient data meta-analysis of a complex intervention. Stroke 41, 2632–2636 10.1161/STROKEAHA.110.588244 20947855

[B19] Dafotakis M. Grefkes C. Eickhoff S. B. Karbe H. Fink G. R. Nowak D. A. (2008). Effects of rTMS on grip force control following subcortical stroke. Exp. Neurol. 211, 407–412 10.1016/j.expneurol.2008.02.018 18395715

[B20] Decarli C. Kawas C. Morrison J. H. Reuter-Lorenz P. A. Sperling R. A. Wright C. B. (2012). Session II: mechanisms of age-related cognitive change and targets for intervention: neural circuits, networks, and plasticity. J. Gerontol. A Biol. Sci. Med. Sci. 67, 747–753 10.1093/gerona/gls111 22570135 PMC3732094

[B21] Dell’osso B. Camuri G. Castellano F. Vecchi V. Benedetti M. Bortolussi S. (2011). Meta-review of metanalytic studies with repetitive transcranial magnetic stimulation (rTMS) for the treatment of major depression. Clin. Pract. Epidemiol. Ment. Health 7, 167–177 10.2174/1745017901107010167 22135698 PMC3227860

[B22] Edwards D. Fregni F. (2008). Modulating the healthy and affected motor cortex with repetitive transcranial magnetic stimulation in stroke: development of new strategies for neurorehabilitation. Neurorehabilitation 23, 3–14 18356585

[B23] Egger M. Davey Smith G. Schneider M. Minder C. (1997). Bias in meta-analysis detected by a simple, graphical test. BMJ 315, 629–634 10.1136/bmj.315.7119.1371 9310563 PMC2127453

[B24] Emara T. H. Moustafa R. R. Elnahas N. M. Elganzoury A. M. Abdo T. A. Mohamed S. A. (2010). Repetitive transcranial magnetic stimulation at 1 Hz and 5 Hz produces sustained improvement in motor function and disability after ischaemic stroke. Eur. J. Neurol. 17, 1203–1209 10.1111/j.1468-1331.2010.03000.x 20402755

[B25] Filippi M. Rocca M. A. Mezzapesa D. M. Ghezzi A. Falini A. Martinelli V. (2004). Simple and complex movement-associated functional MRI changes in patients at presentation with clinically isolated syndromes suggestive of multiple sclerosis. Hum. Brain Mapp. 21, 108–117 10.1002/hbm.10160 14755598 PMC6872084

[B26] Fox M. D. Halko M. A. Eldaief M. C. Pascual-Leone A. (2012). Measuring and manipulating brain connectivity with resting state functional connectivity magnetic resonance imaging (fcMRI) and transcranial magnetic stimulation (TMS). Neuroimage 62, 2232–2243 10.1016/j.neuroimage.2012.01.103 22465297 PMC3518426

[B27] Frank E. Schecklmann M. Landgrebe M. Burger J. Kreuzer P. Poeppl T. B. (2012). Treatment of chronic tinnitus with repeated sessions of prefrontal transcranial direct current stimulation: outcomes from an open-label pilot study. J. Neurol. 259, 327–333 10.1007/s00415-011-6189-4 21808984

[B28] Fregni F. Boggio P. S. Mansur C. G. Wagner T. Ferreira M. J. Lima M. C. (2005). Transcranial direct current stimulation of the unaffected hemisphere in stroke patients. Neuroreport 16, 1551–1555 10.1097/01.wnr.0000177010.44602.5e 16148743

[B29] Fregni F. Boggio P. S. Valle A. C. Rocha R. R. Duarte J. Ferreira M. J. (2006). A sham-controlled trial of a 5-day course of repetitive transcranial magnetic stimulation of the unaffected hemisphere in stroke patients. Stroke 37, 2115–2122 10.1161/01.STR.0000231390.58967.6b 16809569

[B30] Fumagalli M. Vergari M. Pasqualetti P. Marceglia S. Mameli F. Ferrucci R. (2010). Brain switches utilitarian behavior: does gender make the difference? PLoS ONE 5, e8865 10.1371/journal.pone.0008865 20111608 PMC2810338

[B31] Funke K. Benali A. (2011). Modulation of cortical inhibition by rTMS – findings obtained from animal models. J. Physiol. (Lond.) 589(Pt 18), 4423–4435 21768267 10.1113/jphysiol.2011.206573 PMC3208216

[B32] Galea J. M. Vazquez A. Pasricha N. de Xivry J. J. Celnik P. (2011). Dissociating the roles of the cerebellum and motor cortex during adaptive learning: the motor cortex retains what the cerebellum learns. Cereb. Cortex 21, 1761–1770 10.1093/cercor/bhq246 21139077 PMC3138512

[B33] Gao F. Wang S. Guo Y. Wang J. Lou M. Wu J. (2010). Protective effects of repetitive transcranial magnetic stimulation in a rat model of transient cerebral ischaemia: a microPET study. Eur. J. Nucl. Med. Mol. Imaging 37, 954–961 10.1007/s00259-009-1342-3 20107794

[B34] Grefkes C. Nowak D. A. Wang L. E. Dafotakis M. Eickhoff S. B. Fink G. R. (2010). Modulating cortical connectivity in stroke patients by rTMS assessed with fMRI and dynamic causal modeling. Neuroimage 50, 233–242 10.1016/j.neuroimage.2009.12.029 20005962 PMC8020334

[B35] Haaland K. Y. Delaney H. D. (1981). Motor deficits after left or right hemisphere damage due to stroke or tumor. Neuropsychologia 19, 17–27 10.1016/0028-3932(81)90040-3 6785661

[B36] Haaland K. Y. Harrington D. L. (1989). Hemispheric control of the initial and corrective components of aiming movements. Neuropsychologia 27, 961–969 10.1016/0028-3932(89)90071-7 2771034

[B37] Hadley D. Anderson B. S. Borckardt J. J. Arana A. Li X. Nahas Z. (2011). Safety, tolerability, and effectiveness of high doses of adjunctive daily left prefrontal repetitive transcranial magnetic stimulation for treatment-resistant depression in a clinical setting. J. ECT 27, 18–25 10.1097/YCT.0b013e3181ce1a8c 21343710

[B38] Hankey G. J. Jamrozik K. Broadhurst R. J. Forbes S. Anderson C. S. (2002). Long-term disability after first-ever stroke and related prognostic factors in the Perth Community Stroke Study, 1989–1990. Stroke 33, 1034–1040 10.1161/01.STR.0000016922.22707.27 11935057

[B39] Harris J. A. Clifford C. W. Miniussi C. (2008). The functional effect of transcranial magnetic stimulation: signal suppression or neural noise generation? J. Cogn. Neurosci. 20, 734–740 10.1162/jocn.2008.20509 18052790

[B40] Haslinger B. Erhard P. Weilke F. Ceballos-Baumann A. O. Bartenstein P. Grafin von Einsiedel H. (2002). The role of lateral premotor-cerebellar-parietal circuits in motor sequence control: a parametric fMRI study. Brain Res. Cogn. Brain Res. 13, 159–168 10.1016/S0926-6410(01)00104-5 11958958

[B41] Hellmann J. Juttner R. Roth C. Bajbouj M. Kirste I. Heuser I. (2012). Repetitive magnetic stimulation of human-derived neuron-like cells activates cAMP-CREB pathway. Eur. Arch. Psychiatry Clin. Neurosci. 262, 87–91 10.1007/s00406-011-0217-3 21562895

[B42] Hesse S. Waldner A. Mehrholz J. Tomelleri C. Pohl M. Werner C. (2011). Combined transcranial direct current stimulation and robot-assisted arm training in subacute stroke patients: an exploratory, randomized multicenter trial. Neurorehabil. Neural Repair 25, 838–846 10.1177/1545968311413906 21825004

[B43] Hesse S. Werner C. Schonhardt E. M. Bardeleben A. Jenrich W. Kirker S. G. (2007). Combined transcranial direct current stimulation and robot-assisted arm training in subacute stroke patients: a pilot study. Restor. Neurol. Neurosci. 25, 9–15 17473391

[B44] Hummel F. Celnik P. Giraux P. Floel A. Wu W. H. Gerloff C. (2005). Effects of non-invasive cortical stimulation on skilled motor function in chronic stroke. Brain 128(Pt 3), 490–499 10.1093/brain/awh369 15634731

[B45] Hummel F. C. Cohen L. G. (2006). Non-invasive brain stimulation: a new strategy to improve neurorehabilitation after stroke? Lancet Neurol. 5, 708–712 10.1016/S1474-4422(06)70525-7 16857577

[B46] Ipek M. Hilal H. Nese T. Aynur M. Gazanfer E. (2011). Neuronal plasticity in a case with total hemispheric lesion. J. Med. Life 4, 291–294 22567054 PMC3168814

[B47] Ivey F. M. Hafer-Macko C. E. Macko R. F. (2006). Exercise rehabilitation after stroke. NeuroRx 3, 439–450 10.1016/j.nurx.2006.07.011 17012057 PMC3593406

[B48] Jadad A. R. Moore R. A. Carroll D. Jenkinson C. Reynolds D. J. Gavaghan D. J. (1996). Assessing the quality of reports of randomized clinical trials: is blinding necessary? Control Clin. Trials 17, 1–12 10.1016/0197-2456(95)00134-4 8721797

[B49] Kakuda W. Abo M. Kaito N. Ishikawa A. Taguchi K. Yokoi A. (2010a). Six-day course of repetitive transcranial magnetic stimulation plus occupational therapy for post-stroke patients with upper limb hemiparesis: a case series study. Disabil. Rehabil. 32, 801–807 10.3109/09638280903295474 20367405

[B50] Kakuda W. Abo M. Kobayashi K. Momosaki R. Yokoi A. Fukuda A. (2010b). Low-frequency repetitive transcranial magnetic stimulation and intensive occupational therapy for poststroke patients with upper limb hemiparesis: preliminary study of a 15-day protocol. Int. J. Rehabil. Res. 33, 339–345 10.1097/MRR.0b013e32833cdf10 20613547

[B51] Kakuda W. Abo M. Kobayashi K. Momosaki R. Yokoi A. Fukuda A. (2011a). Combination treatment of low-frequency rTMS and occupational therapy with levodopa administration: an intensive neurorehabilitative approach for upper limb hemiparesis after stroke. Int. J. Neurosci. 121, 373–378 10.3109/00207454.2011.560314 21426243

[B52] Kakuda W. Abo M. Kobayashi K. Momosaki R. Yokoi A. Fukuda A. (2011b). Anti-spastic effect of low-frequency rTMS applied with occupational therapy in post-stroke patients with upper limb hemiparesis. Brain Inj. 25, 496–502 10.3109/02699052.2011.608212 21456998

[B53] Kakuda W. Abo M. Kobayashi K. Momosaki R. Yokoi A. Fukuda A. (2011c). Application of combined 6-Hz primed low-frequency rTMS and intensive occupational therapy for upper limb hemiparesis after stroke. Neurorehabilitation 29, 365–371 22207064 10.3233/NRE-2011-0714

[B54] Kakuda W. Abo M. Kobayashi K. Takagishi T. Momosaki R. Yokoi A. (2011d). Baseline severity of upper limb hemiparesis influences the outcome of low-frequency rTMS combined with intensive occupational therapy in patients who have had a stroke. PM R 3, 516–522; quiz 522. 10.1016/j.pmrj.2011.02.015 21665163

[B55] Kakuda W. Abo M. Shimizu M. Sasanuma J. Okamoto T. Yokoi A. (2012). A multi-center study on low-frequency rTMS combined with intensive occupational therapy for upper limb hemiparesis in post-stroke patients. J. Neuroeng. Rehabil. 9, 4 10.1186/1743-0003-9-4 22264239 PMC3271959

[B56] Kent T. A. Rutherford D. G. Breier J. I. Papanicoloau A. C. (2009). What is the evidence for use dependent learning after stroke? Stroke 40(3 Suppl), S139–S140 10.1161/STROKEAHA.108.532051 19064775 PMC3086644

[B57] Khedr E. M. Abdel-Fadeil M. R. Farghali A. Qaid M. (2009). Role of 1 and 3 Hz repetitive transcranial magnetic stimulation on motor function recovery after acute ischaemic stroke. Eur. J. Neurol. 16, 1323–1330 10.1111/j.1468-1331.2008.02522.x 19780802

[B58] Khedr E. M. Ahmed M. A. Fathy N. Rothwell J. C. (2005). Therapeutic trial of repetitive transcranial magnetic stimulation after acute ischemic stroke. Neurology 65, 466–468 10.1212/01.wnl.0000173067.84247.36 16087918

[B59] Khedr E. M. Etraby A. E. Hemeda M. Nasef A. M. Razek A. A. (2010). Long-term effect of repetitive transcranial magnetic stimulation on motor function recovery after acute ischemic stroke. Acta Neurol. Scand. 121, 30–37 10.1111/j.1600-0404.2009.01195.x 19678808

[B60] Kilpatrick C. J. Davis S. M. Tress B. M. Rossiter S. C. Hopper J. L. Vandendriesen M. L. (1990). Epileptic seizures in acute stroke. Arch. Neurol. 47, 157–160 10.1001/archneur.1990.00530020053014 2302087

[B61] Kim D. Y. Ku J. Chang W. H. Park T. H. Lim J. Y. Han K. (2010a). Assessment of post-stroke extrapersonal neglect using a three-dimensional immersive virtual street crossing program. Acta Neurol. Scand. 121, 171–177 10.1111/j.1600-0404.2009.01194.x 19839943

[B62] Kim D. Y. Lim J. Y. Kang E. K. You D. S. Oh M. K. Oh B. M. (2010b). Effect of transcranial direct current stimulation on motor recovery in patients with subacute stroke. Am. J. Phys. Med. Rehabil. 89, 879–886 10.1097/PHM.0b013e3181f70aa7 20962598

[B63] Kim Y. H. You S. H. Ko M. H. Park J. W. Lee K. H. Jang S. H. (2006). Repetitive transcranial magnetic stimulation-induced corticomotor excitability and associated motor skill acquisition in chronic stroke. Stroke 37, 1471–1476 10.1161/01.STR.0000244782.76917.87 16675743

[B64] Knops A. Nuerk H. C. Sparing R. Foltys H. Willmes K. (2006). On the functional role of human parietal cortex in number processing: How gender mediates the impact of a ‘virtual lesion’ induced by rTMS. Neuropsychologia 44, 2270–2283 10.1016/j.neuropsychologia.2006.05.011 16828812

[B65] Koganemaru S. Mima T. Thabit M. N. Ikkaku T. Shimada K. Kanematsu M. (2010). Recovery of upper-limb function due to enhanced use-dependent plasticity in chronic stroke patients. Brain 133, 3373–3384 10.1093/brain/awq193 20688810

[B66] Krakauer J. W. (2005). Arm function after stroke: from physiology to recovery. Semin. Neurol. 25, 384–395 10.1055/s-2005-923533 16341995

[B67] Lefaucheur J. P. Andre-Obadia N. Poulet E. Devanne H. Haffen E. Londero A. (2011). French guidelines on the use of repetitive transcranial magnetic stimulation (rTMS): safety and therapeutic indications. Neurophysiol. Clin. 41, 221–295 10.1016/j.neucli.2011.10.062 22153574

[B68] Liepert J. Zittel S. Weiller C. (2007). Improvement of dexterity by single session low-frequency repetitive transcranial magnetic stimulation over the contralesional motor cortex in acute stroke: a double-blind placebo-controlled crossover trial. Restor. Neurol. Neurosci. 25, 461–465 18334764

[B69] Lindenberg R. Renga V. Zhu L. L. Nair D. Schlaug G. (2010). Bihemispheric brain stimulation facilitates motor recovery in chronic stroke patients. Neurology 75, 2176–2184 10.1212/WNL.0b013e318202013a 21068427 PMC3013585

[B70] Lloyd-Jones D. Adams R. Carnethon M. De Simone G. Ferguson T. B. Flegal K. (2009). Heart disease and stroke statistics – 2009 update: a report from the American Heart Association Statistics Committee and Stroke Statistics Subcommittee. Circulation 119, e21–e181 10.1161/CIRCULATIONAHA.108.191261 19075105

[B71] Lo E. H. (2008). A new penumbra: transitioning from injury into repair after stroke. Nat. Med. 14, 497–500 10.1038/nm1735 18463660

[B72] Lomarev M. P. Kim D. Y. Richardson S. P. Voller B. Hallett M. (2007). Safety study of high-frequency transcranial magnetic stimulation in patients with chronic stroke. Clin. Neurophysiol. 118, 2072–2075 10.1016/j.clinph.2007.06.016 17652018

[B73] Lotze M. Markert J. Sauseng P. Hoppe J. Plewnia C. Gerloff C. (2006). The role of multiple contralesional motor areas for complex hand movements after internal capsular lesion. J. Neurosci. 26, 6096–6102 10.1523/JNEUROSCI.4564-05.2006 16738254 PMC6675223

[B74] Luker J. A. Wall K. Bernhardt J. Edwards I. Grimmer-Somers K. A. (2011). Patients’ age as a determinant of care received following acute stroke: a systematic review. BMC Health Serv. Res. 11, 161 10.1186/1472-6963-11-161 21729329 PMC3150246

[B75] Lum P. Reinkensmeyer D. Mahoney R. Rymer W. Z. Burgar C. (2002). Robotic devices for movement therapy after stroke: current status and challenges to clinical acceptance. Top. Stroke Rehabil. 8, 40–53 10.1310/9KFM-KF81-P9A4-5WW0 14523729

[B76] Madhavan S. Weber K. A. II Stinear J. W. (2011). Non-invasive brain stimulation enhances fine motor control of the hemiparetic ankle: implications for rehabilitation. Exp. Brain Res. 209, 9–17 10.1007/s00221-010-2511-0 21170708

[B77] Mahmoudi H. Borhani Haghighi A. Petramfar P. Jahanshahi S. Salehi Z. Fregni F. (2011). Transcranial direct current stimulation: electrode montage in stroke. Disabil. Rehabil. 33, 1383–1388 10.3109/09638288.2010.532283 21110732

[B78] Malcolm M. P. Triggs W. J. Light K. E. Gonzalez Rothi L. J. Wu S. Reid K. (2007). Repetitive transcranial magnetic stimulation as an adjunct to constraint-induced therapy: an exploratory randomized controlled trial. Am. J. Phys. Med. Rehabil. 86, 707–715 10.1097/PHM.0b013e31813e0de0 17709994 PMC2605430

[B79] Mally J. Dinya E. (2008). Recovery of motor disability and spasticity in post-stroke after repetitive transcranial magnetic stimulation (rTMS). Brain Res. Bull. 76, 388–395 10.1016/j.brainresbull.2007.11.019 18502315

[B80] Mansur C. G. Fregni F. Boggio P. S. Riberto M. Gallucci-Neto J. Santos C. M. (2005). A sham stimulation-controlled trial of rTMS of the unaffected hemisphere in stroke patients. Neurology 64, 1802–1804 10.1212/01.WNL.0000161839.38079.92 15911819

[B81] Nahas Z. Li X. Kozel F. A. Mirzki D. Memon M. Miller K. (2004). Safety and benefits of distance-adjusted prefrontal transcranial magnetic stimulation in depressed patients 55–75 years of age: a pilot study. Depress. Anxiety 19, 249–256 10.1002/da.20015 15274174

[B82] Nair D. G. Renga V. Lindenberg R. Zhu L. Schlaug G. (2011). Optimizing recovery potential through simultaneous occupational therapy and non-invasive brain-stimulation using tDCS. Restor. Neurol. Neurosci. 29, 411–420 22124031 10.3233/RNN-2011-0612 PMC4425274

[B83] Noskin O. Krakauer J. W. Lazar R. M. Festa J. R. Handy C. O’Brien K. A. (2008). Ipsilateral motor dysfunction from unilateral stroke: implications for the functional neuroanatomy of hemiparesis. J. Neurol. Neurosurg. Psychiatr. 79, 401–406 10.1136/jnnp.2007.118463 17635970

[B84] Nowak D. A. Grefkes C. Ameli M. Fink G. R. (2009). Interhemispheric competition after stroke: brain stimulation to enhance recovery of function of the affected hand. Neurorehabil. Neural Repair 23, 641–656 10.1177/1545968309336661 19531606

[B85] Nowak D. A. Grefkes C. Dafotakis M. Eickhoff S. Kust J. Karbe H. (2008). Effects of low-frequency repetitive transcranial magnetic stimulation of the contralesional primary motor cortex on movement kinematics and neural activity in subcortical stroke. Arch. Neurol. 65, 741–747 10.1001/archneur.65.6.741 18541794

[B86] Olivo S. A. Macedo L. G. Gadotti I. C. Fuentes J. Stanton T. Magee D. J. (2008). Scales to assess the quality of randomized controlled trials: a systematic review. Phys. Ther. 88, 156–175 10.2522/ptj.20070147 18073267

[B87] Olsen T. S. (2001). Post-stroke epilepsy. Curr. Atheroscler. Rep. 3, 340–344 10.1007/s11883-001-0029-4 11389801

[B88] Parent A. Hazrati L. N. (1995). Functional anatomy of the basal ganglia. I. The cortico-basal ganglia-thalamo-cortical loop. Brain Res. Brain Res. Rev. 20, 91–127 10.1016/0165-0173(94)00008-D 7711769

[B89] Park S. W. Wolf S. L. Blanton S. Winstein C. Nichols-Larsen D. S. (2008). The EXCITE Trial: Predicting a clinically meaningful motor activity log outcome. Neurorehabil. Neural Repair 22, 486–493 10.1177/1545968308316906 18780883 PMC3754439

[B90] Pascual-Leone A. Houser C. M. Reese K. Shotland L. I. Grafman J. Sato S. (1993). Safety of rapid-rate transcranial magnetic stimulation in normal volunteers. Electroencephalogr. Clin. Neurophysiol. 89, 120–130 10.1016/0168-5597(93)90094-6 7683602

[B91] Pinto P. S. Meoded A. Poretti A. Tekes A. Huisman T. A. (2012). The unique features of traumatic brain injury in children. Review of the characteristics of the pediatric skull and brain, mechanisms of trauma, patterns of injury, complications, and their imaging findings – part 2. J. Neuroimaging 22, e18–e41 10.1111/j.1552-6569.2011.00690.x 22303964

[B92] Poldrack R. A. Sabb F. W. Foerde K. Tom S. M. Asarnow R. F. Bookheimer S. Y. (2005). The neural correlates of motor skill automaticity. J. Neurosci. 25, 5356–5364 10.1523/JNEUROSCI.3880-04.2005 15930384 PMC6725010

[B93] Pomeroy V. M. Cloud G. Tallis R. C. Donaldson C. Nayak V. Miller S. (2007). Transcranial magnetic stimulation and muscle contraction to enhance stroke recovery: a randomized proof-of-principle and feasibility investigation. Neurorehabil. Neural Repair 21, 509–517 10.1177/1545968307300418 17409389

[B94] Quintana H. (2005). Transcranial magnetic stimulation in persons younger than the age of 18. J. ECT 21, 88–95 10.1097/01.yct.0000162556.02720.58 15905749

[B95] Rachid F. Bertschy G. (2006). Safety and efficacy of repetitive transcranial magnetic stimulation in the treatment of depression: a critical appraisal of the last 10 years. Neurophysiol. Clin. 36, 157–183 10.1016/j.neucli.2006.08.006 17046610

[B96] Ratan R. R. Noble M. (2009). Novel multi-modal strategies to promote brain and spinal cord injury recovery. Stroke 40(3 Suppl), S130–S132 10.1161/STROKEAHA.108.534933 19064774 PMC2655641

[B97] Reith J. Jorgensen H. S. Nakayama H. Raaschou H. O. Olsen T. S. (1997). Seizures in acute stroke: predictors and prognostic significance. The Copenhagen Stroke Study. Stroke 28, 1585–1589 10.1161/01.STR.28.8.1585 9259753

[B98] Richards L. Gonzalez Rothi L. J. Davis S. Wu S. S. Nadeau S. E. (2006). Limited dose response to constraint-induced movement therapy in patients with chronic stroke. Clin. Rehabil. 20, 1066–1074 10.1177/0269215506071263 17148518

[B99] Richards L. G. Stewart K. C. Woodbury M. L. Senesac C. Cauraugh J. H. (2008). Movement-dependent stroke recovery: a systematic review and meta-analysis of TMS and fMRI evidence. Neuropsychologia 46, 3–11 10.1016/j.neuropsychologia.2007.08.013 17904594 PMC2248459

[B100] Rossi S. Hallett M. Rossini P. M. Pascual-Leone A. (2009). Safety, ethical considerations, and application guidelines for the use of transcranial magnetic stimulation in clinical practice and research. Clin. Neurophysiol. 120, 2008–2039 10.1016/j.clinph.2009.08.016 19833552 PMC3260536

[B101] Rouiller E. M. Yu X. H. Moret V. Tempini A. Wiesendanger M. Liang F. (1998). Dexterity in adult monkeys following early lesion of the motor cortical hand area: the role of cortex adjacent to the lesion. Eur. J. Neurosci. 10, 729–740 10.1046/j.1460-9568.1998.00075.x 9749734

[B102] Ryan S. Bonilha L. Jackson S. R. (2006). Individual variation in the location of the parietal eye fields: a TMS study. Exp. Brain Res. 173, 389–394 10.1007/s00221-006-0379-9 16506006

[B103] Sangvatanakul P. Hillege S. Lalor E. Levi C. Hill K. Middleton S. (2010). Setting stroke research priorities: the consumer perspective. J. Vasc. Nurs. 28, 121–131 10.1016/j.jvn.2010.09.001 21074114

[B104] Sasaki N. Mizutani S. Kakuda W. Abo M. (2011). Comparison of the effects of high- and low-frequency repetitive transcranial magnetic stimulation on upper limb hemiparesis in the early phase of stroke. J. Stroke Cerebrovasc. Dis. 10.1016/j.jstrokecerebrovasdis.2011.10.004 22177936

[B105] Schmahmann J. D. Pandya D. N. (1997). The cerebrocerebellar system. Int. Rev. Neurobiol. 41, 31–60 10.1016/S0074-7742(08)60338-4 9378595

[B106] Schwarzkopf D. S. Silvanto J. Rees G. (2011). Stochastic resonance effects reveal the neural mechanisms of transcranial magnetic stimulation. J. Neurosci. 31, 3143–3147 10.1523/JNEUROSCI.4863-10.2011 21368025 PMC3059801

[B107] Shimizu T. Hosaki A. Hino T. Sato M. Komori T. Hirai S. (2002). Motor cortical disinhibition in the unaffected hemisphere after unilateral cortical stroke. Brain 125(Pt 8), 1896–1907 10.1093/brain/awf183 12135979

[B108] So E. L. Annegers J. F. Hauser W. A. O’Brien P. C. Whisnant J. P. (1996). Population-based study of seizure disorders after cerebral infarction. Neurology 46, 350–355 10.1212/WNL.46.2.350 8614493

[B109] Sohn Y. H. Jung H. Y. Kaelin-Lang A. Hallett M. (2002). Effect of levetiracetam on rapid motor learning in humans. Arch. Neurol. 59, 1909–1912 10.1001/archneur.59.12.1909 12470179

[B110] Stagg C. J. Bachtiar V. O’Shea J. Allman C. Bosnell R. A. Kischka U. (2012). Cortical activation changes underlying stimulation-induced behavioural gains in chronic stroke. Brain 135(Pt 1), 276–284 10.1093/brain/awr313 22155982 PMC3267983

[B111] Takeuchi N. Chuma T. Matsuo Y. Watanabe I. Ikoma K. (2005). Repetitive transcranial magnetic stimulation of contralesional primary motor cortex improves hand function after stroke. Stroke 36, 2681–2686 10.1161/01.STR.0000189658.51972.34 16254224

[B112] Takeuchi N. Tada T. Toshima M. Chuma T. Matsuo Y. Ikoma K. (2008). Inhibition of the unaffected motor cortex by 1 Hz repetitive transcranical magnetic stimulation enhances motor performance and training effect of the paretic hand in patients with chronic stroke. J. Rehabil. Med. 40, 298–303 10.2340/16501977-0181 18382826

[B113] Takeuchi N. Tada T. Toshima M. Matsuo Y. Ikoma K. (2009). Repetitive transcranial magnetic stimulation over bilateral hemispheres enhances motor function and training effect of paretic hand in patients after stroke. J. Rehabil. Med. 41, 1049–1054 10.2340/16501977-0454 19894000

[B114] Tanaka S. Takeda K. Otaka Y. Kita K. Osu R. Honda M. (2011). Single session of transcranial direct current stimulation transiently increases knee extensor force in patients with hemiparetic stroke. Neurorehabil. Neural Repair 25, 565–569 10.1177/1545968311402091 21436391

[B115] Valero-Cabre A. Payne B. R. Pascual-Leone A. (2007). Opposite impact on 14C-2-deoxyglucose brain metabolism following patterns of high and low frequency repetitive transcranial magnetic stimulation in the posterior parietal cortex. Exp. Brain Res. 176, 603–615 10.1007/s00221-006-0639-8 16972076

[B116] Wagner T. Eden U. Fregni F. Valero-Cabre A. Ramos-Estebanez C. Pronio-Stelluto V. (2008). Transcranial magnetic stimulation and brain atrophy: a computer-based human brain model study. Exp. Brain Res. 186, 539–550 10.1007/s00221-007-1258-8 18193208 PMC3374637

[B117] Ward N. S. Brown M. M. Thompson A. J. Frackowiak R. S. (2003). Neural correlates of motor recovery after stroke: a longitudinal fMRI study. Brain 126(Pt 11), 2476–2496 10.1093/brain/awg245 12937084 PMC3717457

[B118] Werhahn K. J. Conforto A. B. Kadom N. Hallett M. Cohen L. G. (2003). Contribution of the ipsilateral motor cortex to recovery after chronic stroke. Ann. Neurol. 54, 464–472 10.1002/ana.10686 14520658

[B119] Williams J. A. Pascual-Leone A. Fregni F. (2010). Interhemispheric modulation induced by cortical stimulation and motor training. Phys. Ther. 90, 398–410 10.2522/ptj.20090075 20110339

[B120] Wong S. S. Wilczynski N. L. Haynes R. B. (2006). Comparison of top-performing search strategies for detecting clinically sound treatment studies and systematic reviews in MEDLINE and EMBASE. J. Med. Libr. Assoc. 94, 451–455 17082841 PMC1629423

[B121] Yoon K. J. Lee Y. T. Han T. R. (2011). Mechanism of functional recovery after repetitive transcranial magnetic stimulation (rTMS) in the subacute cerebral ischemic rat model: neural plasticity or anti-apoptosis? Exp. Brain Res. 214, 549–556 10.1007/s00221-011-2853-2 21904929

[B122] Yozbatiran N. Alonso-Alonso M. See J. Demirtas-Tatlidede A. Luu D. Motiwala R. R. (2009). Safety and behavioral effects of high-frequency repetitive transcranial magnetic stimulation in stroke. Stroke 40, 309–312 10.1161/STROKEAHA.108.522144 18845801 PMC3366156

