# Supplementary material for: Systematic Review of Parameters of Stimulation, Clinical Trial Design Characteristics, and Motor Outcomes in Non-Invasive Brain Stimulation in Stroke
Source: Front Psychiatry. 2012 Nov 12;3:88. doi: 10.3389/fpsyt.2012.00088 (PMC3495265; doi:10.3389/fpsyt.2012.00088)
Supplement: Supplementary Table S1 — Adverse events and safety outcome for selected peer-reviewed articles. MMSE, mini-mental state examination; EEG, electroencephalography. [file 30952_Fregni_DataSheet1.DOCX]

|  | **Safety outcome** | | | | | **Exclusion/Inclusion presumptively related to safety** | | | | | | |
| --- | --- | --- | --- | --- | --- | --- | --- | --- | --- | --- | --- | --- |
|  | **Adverse events** | **Cognitive** | **Mood** | **EEG** | **Others** | **Medication as exclusion criteria** | **Limited by Age** | **Cognition** | **Mood** | **Seizure / Epilepsy / EEG** | **Comorbidity** | **Others** |
| **Fregni et al. 2006** | One patient reported a mild headache. One patient reported an increase in anxiety. In the sham rTMS group, one patient reported an increase in the tiredness and one patient reported a mild headache | Stroop test, Digit Span and MMSE no significant effect of time, group, or interaction time*group. | Not mentioned | No changes pre and post stimulation. | Not mentioned | Not mentioned | Not mentioned | No other neuropysichiatric comorbidity | No other neuropysichiatric comorbidity | No other neuropysichiatric comorbidity | No clinically significant or unstable medical disorder, with a history of substance abuse. | Not mentioned |
| **Takeuchi et al. 2005** | Subjects did not report any adverse side effects | Not mentioned | Not mentioned | Not mentioned | Not mentioned | Not mentioned | Not mentioned | Normal Mini-Mental State Examination score | Not mentioned | Not mentioned | Not mentioned | Not mentioned |
| **Mansur et al. 2005** | Not mentioned | Not mentioned | Not mentioned | Not mentioned | Not mentioned | Not mentioned | Not mentioned | Not mentioned | Not mentioned | Not mentioned | Not mentioned | Not mentioned |
| **Werhahn et al. 2003** | Not mentioned | Not mentioned | Not mentioned | Not mentioned | Not mentioned | Not mentioned | Not mentioned | Inclusion normal mini-mental | Not mentioned | Not mentioned | Not mentioned | Not mentioned |
| **Nowak et al. 2008** | Not mentioned | Not mentioned | Not mentioned | Not mentioned | Not mentioned | Not mentioned | Not mentioned | Score of less than 24 points on the Folstein Mini Mental Status Examination, | Absence of relevant depression as suggested by a score of less than 6 points on the Hamilton Depression Scale | Not mentioned | Not mentioned | Not mentioned |
| **Takeuchi et al. 2008** | Did not report any adverse effects | Not mentioned | Not mentioned | Not mentioned | Not mentioned | Not mentioned | Not mentioned | Inclusion normal mini-mental | Not mentioned | No history of seizure | No severe internal carotid artery stenosis | No intracranial metallic implant |
| **Dafotakis et al. 2008** | Not mentioned | Not mentioned | Not mentioned | Not mentioned | Not mentioned | Not being treated with spasmolytic drugs. | No history suggested coexistent neurological illness | Included patients with score of >24 points on the Folstein’s Mini Mental Status Examination | Included patients with absence of a relevant depression as suggested by a score of less than 6 points on the Hamilton Depression Scale | No history of seizure | No medical or orthopedic illness of the upper extremities, psychiatric illness. | No pathological conditions referred to as contraindications for rTMS in the guidelines suggested by Wasserman (e.g. cardiac pacemakers, intracranial implants, implanted medication pumps, epilepsy, pregnancy) |
| **Kakuda et al. 2010** | None showed any adverse effects | Not mentioned | Not mentioned | Not mentioned | Not mentioned | Not mentioned | between 18 and 80 years; | No cognitive impairment (a pretreatment Mini Mental State Examination Score of 26) | No active physical or mental illness requiring medical management | No history of seizure, no documented epileptic discharge on the pretreatment electroencephalogram | No pathological conditions referred to as contraindications for rTMS in the guidelines suggested by Wasserman; | Not mentioned |
| **Liepert et al. 2007** | Not mentioned | Not mentioned | Not mentioned | Not mentioned | Not mentioned | Exclude patient taking drugs known to interfere with brain excitability | Not mentioned | Exclude dementia | Not mentioned | No other neurological diseases apart from the stroke | pregnancy | No heart pacemakers and metallic objects in the brain |
| **Grefkes et al. 2010** | Not mentioned | Not mentioned | Not mentioned | Not mentioned | Not mentioned | Not mentioned | Not mentioned | Not mentioned | Not mentioned | Not mentioned | Not mentioned | Not mentioned |
| **Lotze et al. 2006** | Not mentioned | Not mentioned | Not mentioned | Not mentioned | Not mentioned | Not mentioned | Not mentioned | No other neurological illness | No psychiatric illness | No other neurological illness | Not mentioned | Not mentioned |
| **Lomarev et al. 2007** | Two dropouts included a woman with a high MT (89% of TMS machine output) in whom a single applied rTMS train at 100% machine output was not tolerated. | Not mentioned | Not mentioned | Not mentioned | Brief EMG bursts, possibly peripheral manifestations of after discharges, and spread of excitation to proximal muscles. Considered to be associated with a high risk of seizure occurrence. | Exclude patient taking psychotropic medication or drugs reducing seizure threshold | Not mentioned | Not mentioned | Not mentioned | Exclude patients with an episode of seizure or epileptiform electroencephalographic (EEG) activity | With a history of more than one stroke | Having any metal implants in the head |

|  | **Safety outcome** | | | | | **Exclusion/Inclusion presumptively related to safety** | | | | | | |
| --- | --- | --- | --- | --- | --- | --- | --- | --- | --- | --- | --- | --- |
|  | **Adverse events** | **Cognitive** | **Mood** | **EEG** | **Others** | **Medication as exclusion criteria** | **Limited by Age** | **Cognition** | **Mood** | **Seizure / Epilepsy / EEG** | **Comorbidity** | **Others** |
| Malcolm et al. 2007 | There were no discernible adverse effects | Not mentioned | Not mentioned | Not mentioned | Not mentioned | Exclude: drugs that might potentially inhibit neuroplasticity (narcoleptics, -1 noradrenergic antagonists, -2 noradrenergic agonists, anticonvulsants, benzodiazepines, anticholinergics, and tricyclic antidepressants); use of medications that may lower seizure thresh- old (e.g., Metronidazole) | Not mentioned | Exclude dementia and mental retardation. | Exclude patients with major psychiatric illness | Exclude patients with history of epilepsy and evidence of epileptiform activity on electroencephalography obtained before beginning treatment | Exclude patients with history of brain tumor, learning disorder, drug or alcohol abuse or major head trauma; history or radiographic evidence of arteriovenous malformation, intracortical hemorrhage, subarachnoid hemorrhage, or bilateral cerebrovascular disease. | Pregnancy; history of implanted pacemaker or medication pump, metal plate in skull, or metal objects in the eye or skull |
| Khedr et al. 2005 | no side effects of rTMS except occasional mild headache. | Not mentioned | Not mentioned | Not mentioned | Not mentioned | previous administration of tranquilizer | Not mentioned | Exclude patient with neurologic disease other than stroke | Not mentioned | Exclude patient with neurologic disease other than stroke | Unstable cardiac dysrhythmia, head injury | Not mentioned |
| Khedr et al. 2010 | Our results are supported by Fregni et al. (7) they confirmed that increased dose of rTMS is not associated with cognitive adverse effects and ⁄ or epileptogenic activity. | not associated with cognitive adverse effect | Not mentioned | No focal or generalized sharp activity or epileptic activity (spike wave complex) could be recorded before and after the rTMS sessions. | Not mentioned | Exclude patient with previous administration of tranquilize | Not mentioned | Exclude patient with neurologic disease other than stroke | Not mentioned | Exclude patient with epilepsy | Exclude patient with extensive infarction, severe flaccid hemiplegia, head injury or unstable cardiac arrhythmia, previous administration of tranquilizer. | Exclude patients with intracranial metallic, magnetic pieces, with pacemakers or any other device. |
| Yozbatiran et al. 2009 | No witnessed adverse events during rTMS, and patients reported no new symptoms through Visit 3. Systolic blood pressure, assessed in the first minute after rTMS, increased 7 mm Hg as compared with immediately pre-rTMS (P<0.05, Table 2), a change accounted for by 9 of 12 patients, but no such change was apparent for diastolic blood pressure or pulse. None of the behavioral measures showed a decrement. | Not mentioned | Not mentioned | Not mentioned | Not mentioned | Not mentioned | Not mentioned | Exclude patients with coexistent neurological disease | Exclude patients with coexistent psychiatric disease and Zung Depression score >=50. | No history of seizure | Exclusion patients with pre-stroke Rankin score >1; other focal cortical pathology; decreased alertness, language reception, or attention; advanced systemic disease; terminal illness; prior TMS; and TMS/MRI contra- indication | pregnant/ lactating |
| Ameli et al. 2009 | Not mentioned | Not mentioned | Not mentioned | Not mentioned | Not mentioned | Limitation of the study is intake of medication, which, among others, could influence cortical excitability. | Not mentioned | Mini-mental normal | No psychiatric disease and absence of a relevant depression as indexed by a score of 18 points on the Beck Depression Inventory | No coexistent general neurological illness | No coexistent medical, or orthopedic illness; | Not mentioned |
| Hummel et al. 2006 | Not mentioned | Not mentioned | Not mentioned | Not mentioned | Not mentioned | Not mentioned | Not mentioned | Exclude patients serious cognitive deficits (MMSE <23/30 points | Exclude patient with severe depression | Not mentioned | Exclude patients with severe language disturbances, history of severe alcohol or drug abuse, | Not mentioned |

|  | **Safety outcome** | | | | | **Exclusion/Inclusion presumptively related to safety** | | | | | | |
| --- | --- | --- | --- | --- | --- | --- | --- | --- | --- | --- | --- | --- |
|  | **Adverse events** | **Cognitive** | **Mood** | **EEG** | **Others** | **Medication as exclusion criteria** | **Limited by Age** | **Cognition** | **Mood** | **Seizure / Epilepsy / EEG** | **Comorbidity** | **Others** |
| Hesse et al. 2007 | No persistent side effects occurred; four patients noticed a slight itching under the electrodes and two subjects a bearable headache in the first week immediately following tDCS. No major side effects | Not mentioned | Not mentioned | Not mentioned | Not mentioned | Exclude medications altering the level of cortical excitability (e.g. antiepileptics, neuroleptics, benzodiazepines, antidepressants), and medications with a presumed positive or negative effect on brain plasticity (e.g. Dopamine, Fluoxetine, D-Amphetamine). | Age < 80 years | Exclude patients with severe cognitive impairment | Not mentioned | Excluded patient with EEG suspect of elevated cortical excitability and preceding epileptic fits | Not mentioned | Sensitive scalp skin; Metallic implants within the brain; Previous brain neurosurgery; |
| Fregni et al. 2005 | All patients tolerated tDCS well and no adverse effects related to the application of tDCS were observed. | No effects on attention or fatigue | Not mentioned | Not mentioned | Not mentioned | Not mentioned | Not mentioned | Not mentioned | Not mentioned | Not mentioned | Not mentioned | Not mentioned |
| Emara et al. 2010 | Not mentioned | No significant change in MMSE scores was observed between | Not mentioned | Not mentioned | Not mentioned | Not mentioned | Not mentioned | Exclude cognitive impairment [mini-mental state examination (MMSE) score <24 | Not mentioned | No history of seizures | Excluded if they had severe hand weakness, receptive dysphasia, hemineglect, anosognosia, hemorrhage and other non-vascular pathology | Excluded if they had implanted pace makers or other metal implants. |
| Boggio et al. 2007 | There were no adverse effects associated with application of (weekly or daily consecutive) repeated sessions of tDCS | Not mentioned | Not mentioned | Not mentioned | Not mentioned | Not mentioned | Not mentioned | Not mentioned | Not mentioned | Not mentioned | Not mentioned | Not mentioned |
| (Pomeroy et al. 2007 | One subject in the present study reported an adverse effect of rTMS (headache) | Not mentioned | Not mentioned | Not mentioned | Not mentioned | Not mentioned | Not mentioned | Not mentioned | Not mentioned | No history of seizures | Exclude for safety reasons, the infarct was limited to 50% of MCA territory | Who had no contraindications to rTMS (no implanted metal, no implanted electronic devices) |
| Lindenberg et al. 2010 | No adverse effects were observed by the investigators or reported by the patients except a mild tingling sensation at the site of the electrodes at the beginning of the stimulation, which is a common finding across different studies | Not mentioned | Not mentioned | Not mentioned | Not mentioned | No concurrent use of CNS-affecting drugs. | Not mentioned | No additional neurologic or psychiatric disorders | No additional neurologic or psychiatric disorders | No additional neurologic or psychiatric disorders | Not mentioned | Not mentioned |
| Chang et al. 2010 | no adverse side –effect | Not mentioned | Not mentioned | Not mentioned | Not mentioned | Not mentioned | Not mentioned | Not mentioned | Exclude patients with any neuropsychiatric comorbidity other than stroke, | No history of seizure | Exclude patients with direct injury to the primary motor cortex, complete internal carotid artery occlusion. | No intracranial metallic implant. |
| Kim et al. 2010 | 2 dropout : Headache &_dizziness. No other subjects reported adverse effects. | Not mentioned | Not mentioned | Not mentioned | Not mentioned | Patients who were taking Na􏰁 or Ca2􏰁 channel blockers or N-methyl-D-aspartate (NMDA) receptor antagonists were also excluded. | Not mentioned | Exclude patients with depression | Exclude patients with cognitive deficits (based on the mini-mental state examination 10 of 30 points) | No history of seizure | Exclude patients with cerebellar or brain stem lesions; unstable medical condition, severe language disturbance, neglect, history of severe alcohol or drug abuse, previous stroke that resulted in residual disability, pre morbid arm impairment, and hemiplegic shoulder pain. | Exclude patients with presence of a metallic foreign body implant, such as a pacemaker or artificial cochlea; |

|  | **Safety outcome** | | | | | **Exclusion/Inclusion presumptively related to safety** | | | | | | |
| --- | --- | --- | --- | --- | --- | --- | --- | --- | --- | --- | --- | --- |
|  | **Adverse events** | **Cognitive** | **Mood** | **EEG** | **Others** | **Medication as exclusion criteria** | **Limited by Age** | **Cognition** | **Mood** | **Seizure / Epilepsy / EEG** | **Comorbidity** | **Others** |
| Kakuda et al. 2011 | Not mentioned | Not mentioned | Not mentioned | Not mentioned | Not mentioned | Not mentioned | Age at intervention between 18–80 years | No cognitive impairment with a pre-treatment Mini Mental State Examination score of more than 26; | No active mental illness requiring medical management | No recent history of seizure and no documentation of epileptic discharge on pretreatment electroencephalogram | No active physical illness requiring medical management | No contraindications for rTMS as set in the guidelines suggested by Wassermann (e.g. cardiac pace- makers, intracranial implants, implanted medication pumps, pregnancy) |
| Kakuda et al. 2010 | No side effect | no cognitive deficit | Not mentioned | Not mentioned | Not mentioned | Not mentioned | Age 18-80 | Not mentioned | Not mentioned | exclude epileptic discharge | Not mentioned | no history of nerve block |
| Koganemaru et al. 2010 | Not mentioned | Not mentioned | Not mentioned | Not mentioned | Not mentioned | antispastic medications or other drugs that could interfere with cortical excitability. | Not mentioned | Not mentioned | Not mentioned | Not mentioned | Not mentioned | Not mentioned |
| Richards et al. 2006 | No subjects experienced side effects during the experiments. | Not mentioned | Not mentioned | Not mentioned | Not mentioned | use of no more than one medication that had the potential for inhibiting neuroplasticity (e.g. neuroleptics, a-1 noradrenergic antagonists or a-2 noradenergic agonists, benzodiazepines, anticonvulsants, or tricyclic antidepressants). | Not mentioned | No history of other neurological conditions | No history of other psychiatric conditions | No history of other neurological conditions | Not mentioned | Not mentioned |
| Kim et al. 2006 | no participant reported any adverse effect or seizures. | Not mentioned | Not mentioned | Not mentioned | Not mentioned | Not mentioned | Not mentioned | Not mentioned | Not mentioned | No history of seizure | No severe internal carotid artery stenosis or direct damage to the primary motor cortex | No intracranial metallic implant. |
| Khedr et al. 2009 | All the patients tolerated rTMS well without any adverse effects. | Not mentioned | Not mentioned | Not mentioned | Not mentioned | No previous administration of tranquilizer | Not mentioned | Exclusion patients with neurological disease other than stroke | Not mentioned | No history of epilepsy | Exclusion patients with extensive infarction, unstable cardiac dysrhythmia, and patients who were unable to give informed consent because of severe aphasia, ansognosia or cognitive deficit | Patients with intracranial metallic, magnetic pieces, with pacemakers or any other device were also excluded |
| Takeuchi et al. 2009 | The subjects did not report any adverse side-effects during the course of the study | Not mentioned | Not mentioned | Not mentioned | Not mentioned | No specified | Not mentioned | Normal Mini-Mental State Examination score | Not mentioned | No history of seizure | Severe internal carotid artery stenosis | No intracranial metallic implant |
| Mally and Dinya 2008 | Not mentioned | Not mentioned | Not mentioned | Not mentioned | Not mentioned | Only taking drug-controlled hypertension, | Not mentioned | Not mentioned | Not mentioned | Not mentioned | Except for drug-controlled hypertension, they had no other disease. | Not mentioned |
| Kakuda et al. 2011 | No side effect | Not mentioned | Not mentioned | Not mentioned | Not mentioned | no current use of antiepileptic medications for the prevention of seizure | 18-80 years | No cognitive impairment with a pretreatment Mini Mental State Examination score of more than 26 | No active mental illness requiring medical management | No recent history of seizure; No documented epileptic discharges on pretreatment electroencephalogram; | No active physical illness requiring medical management | no pathological conditions known to be contraindications for rTMS in the guidelines suggested by Wassermann |
| Kakuda et al. 2012 | No side effect | Not mentioned | Not mentioned | Not mentioned | Not mentioned | no current use of antiepileptic medications for the prevention of seizure | 18-80 years | No cognitive impairment with a pretreatment Mini Mental State Examination score of more than 26 | No active mental illness requiring medical management | No recent history of seizure; No documented epileptic discharges on pretreatment electroencephalogram; | No active physical illness requiring medical management | no pathological conditions known to be contraindications for rTMS in the guidelines suggested by Wassermann |

|  | **Safety outcome** | | | | | **Exclusion/Inclusion presumptively related to safety** | | | | | | |
| --- | --- | --- | --- | --- | --- | --- | --- | --- | --- | --- | --- | --- |
|  | **Adverse events** | **Cognitive** | **Mood** | **EEG** | **Others** | **Medication as exclusion criteria** | **Limited by Age** | **Cognition** | **Mood** | **Seizure / Epilepsy / EEG** | **Comorbidity** | **Others** |
| Sasaki et al. 2011 | No side effect | Not mentioned | Not mentioned | Not mentioned | Not mentioned | Not mentioned | 45 - 80 years | No disturbance of consciousness; No apparent cognitive deficit | Not mentioned | Not mentioned | No serious general complications requiring intensive medical management (e.g., pneumonia, heart failure, urinary tract infection, or malnutritional state) | no pathologic conditions referred to as contraindications for rTMS in the guidelines |
| Stagg et al. 2012 | Two patients withdrew from the study before completion, one due to claustrophobia and one due to unrelated medical reasons. (Note: Not related with tDCS) | Not mentioned | Not mentioned | Not mentioned | Not mentioned | No patients were on CNS-active medications | Not mentioned | Not mentioned | Not mentioned | Not mentioned | no previous history, signs, or symptoms of other neurological conditions | Not mentioned |
| Kakuda et al. 2011 | No side effect | Not mentioned | Not mentioned | Not mentioned | Not mentioned | no current use of antiepileptic medications for the prevention of seizure | 18-80 years | No cognitive impairment with a pretreatment Mini Mental State Examination score of more than 26 | No active mental illness requiring medical management | No recent history of seizure; No documented epileptic discharges on pretreatment electroencephalogram; | No active physical illness requiring medical management | no pathological conditions known to be contraindications for rTMS in the guidelines suggested by Wassermann |
| Madhavan et al. 2011 | No side effect | Not mentioned | Not mentioned | Not mentioned | Not mentioned | Patients were excluded with medications known to alter central nervous system excitability | Not mentioned | Mini-mental normal | Not mentioned | Patients were excluded with history of seizures | Not mentioned | Patients were excluded with contraindications to magnetic resonance imaging (MRI) or TMS |
| Tanaka et al. 2011 | No side effect | Not mentioned | Not mentioned | Not mentioned | Visual analogue scale (VAS) scores of patients’ attention, fatigue, pain, and discomfort | Not mentioned | Not mentioned | Mini-mental normal | Not mentioned | Not mentioned | Not mentioned | Not mentioned |
| Kakuda et al. 2011 | No side effect | Not mentioned | Not mentioned | Not mentioned | Not mentioned | No active mental illness requiring medical management | 18-80 years | No cognitive impairment with a pretreatment Mini Mental State Examination score of more than 26 | No active mental illness requiring medical management | No recent history of seizure; No documented epileptic discharges on pretreatment electroencephalogram; | No active physical illness requiring medical management | no pathological conditions known to be contraindications for rTMS in the guidelines suggested by Wassermann |
| Avenanti et al. 2012 | Not mentioned | Not mentioned | Not mentioned | EEG monitoring used to exclude patients. No EEG results after the beginning of the study mentioned. | Not mentioned | Not mentioned | Not mentioned | Not mentioned | Not mentioned | Prolonged EEG monitoring used to exclude presence of epileptogenic activity. | Excluded moderate to severe severity or any medically significant comorbidity. | Not mentioned |
| Bolognini et al. 2011 | ­­None of the patients reported any events | Not mentioned | Not mentioned (baseline data) | Not mentioned | Not mentioned | Not mentioned | Not mentioned | Not mentioned | Not mentioned (exclusion criteria were pre-stroke moderate to severe major depression) | Not mentioned | Not mentioned | Not mentioned |

|  | **Safety outcome** | | | | | **Exclusion/Inclusion presumptively related to safety** | | | | | | |
| --- | --- | --- | --- | --- | --- | --- | --- | --- | --- | --- | --- | --- |
|  | **Adverse events** | **Cognitive** | **Mood** | **EEG** | **Others** | **Medication as exclusion criteria** | **Limited by Age** | **Cognition** | **Mood** | **Seizure / Epilepsy / EEG** | **Comorbidity** | **Others** |
| Conforto et al. 2011 | Adverse events were logged per treatment session without any serious intervention-related adverse events. No significant differences in rates of sleepiness, or headache, local or nuchal pain between the groups. (Head or nuchal pain were mild and subsided spontaneously after a few minutes). | None mentioned | None mentioned | Not mentioned (observed by investigator. No seizures or syncope) | Blood pressure and HR (no significant differences). | Excluded "Use of drugs that might interfere on cortical excitability" | Age 18-80 | "inability to provide informed consent due to severe language or cognitive impairment" | Not mentioned | Not mentioned | Excluded "other neurological or severe chronic diseases" | Excluded shoulder pain; joint deformity in the paretic upper limb; |
| Hesse et al. 2011 | Tingling underneath the electrodes (n=12 evenly distributed across 3 groups). Transient headaches (n = 4, in those with previously known history of headaches). | None mentioned | None mentioned | None. Used as exclusion criteria | None mentioned | Excluded anticonvulsant and neuroleptic medications. | Age 18-79 | None mentioned | Not mentioned | Excluded preceding epileptic seizures and EEG with elevated cortical excitability. | No other neurological or orthopedic disease impairing the upper limb. | No sensitive scalp skin, metallic implants within the brain, and previous brain neurosurgery. |
| Mahmoudi et al. 2011 | None mentioned | None mentioned | None mentioned | None mentioned | None mentioned | None mentioned | None mentioned | Not mentioned | Excluded patients with any clinically significant or unstable  psychiatric disorder or substance abuse. | Not mentioned | Excluded any significant or unstable medical or neuropsychiatric comorbidity other than stroke. | Not mentioned |
| Nair et al. 2011 | None mentioned | None mentioned | None mentioned | None mentioned | None mentioned | None of the patients were taking anti-depressant medications or stimulants | Not mentioned | Not mentioned | Not mentioned | Not mentioned | Excluded: Patients with a previous history of stroke, bilateral infarcts, hemorrhage arthritis, chronic pain and other neurological diseases. | Not mentioned |
| Chang et al. 2011 | Not mentioned | Not mentioned | Not mentioned | Not mentioned | Not mentioned | Not mentioned | Not mentioned | Not mentioned | Not mentioned | Excluded post-stroke seizure disorder; and patients with epileptic spikes on EEG. | Not mentioned | Direct injury to the primary motor cortex and carotid artery occlusion on the ipsilesional side. |
| Hummel et al. 2005 | No effect on attention or fatigue. Discomfort/pain was negligible ranging between 1 and 2 out of 10, and it was comparable in the tDCS and Sham sessions. | None mentioned | None mentioned | None mentioned | None mentioned | None mentioned | Not mentioned | Excluded severe cognitive deficits (Mini-Mental Status Examination score <23/30) and severe language disturbances. | Excluded severe depression and history of severe alcohol or drug abuse. | Not mentioned | Not mentioned | Not mentioned |

**Table S1:**

**MMSE**: Mini-Mental State Examination; **EEG**: Electroencephalography
